# Regioselectivity of the alkylation of *S*-substituted 1,2,4-triazoles with dihaloalkanes

**DOI:** 10.1186/s13065-016-0165-0

**Published:** 2016-04-27

**Authors:** Ahmed T. A. Boraei, El Sayed H. El Ashry, Axel Duerkop

**Affiliations:** Chemistry Department, Faculty of Science, Suez Canal University, Ismailia, 41522 Egypt; Chemistry Department, Faculty of Science, Alexandria University, Alexandria, Egypt; Institute of Analytical Chemistry, Chemo and Biosensors, University of Regensburg, Universitätsstrasse 31, 93053 Regensburg, Germany

**Keywords:** 1,2,4-Triazoles, Alkylation, Regioselectivity, Single-crystal X-ray diffraction

## Abstract

**Background:**

1,2,4-Triazole3-thiones are good scaffolds for preparation of new lead compounds. Their derivatives attracted the attention of chemists due to their wide spectrum of biological activities. Alkylsulfanyl-1,2,4-triazoles have three nucleophilic sites (nitrogens) ready for reaction with electrophiles. Herein, new regioselective isomers were synthesized by the reaction of benzylsulfanyl-1,2,4-triazole with various dihaloalkanes. Regioselectivity was determined by X-ray crystallography and NMR.

**Results:**

Coupling of 3-benzylsufanyl-5-(1*H*-indolyl)-1,2,4-triazole with dibromomethane, 1,2-dichloroethane, 1,3-dibromopropane and di(bromomethyl)quinoxaline was investigated in the presence of potassium carbonate in acetone. In the case of dibromomethane three different bis(triazolyl)methane isomers (–*N*^1^–CH_2_–*N*^1^-**4**, –*N*^1^–CH_2_–*N*^2^-**5**, –*N*^2^–CH_2_–*N*^2^-**6**) were formed in which the two bromide atoms were replaced by two triazole moieties. Among these isomers the reaction was regioselective towards the –*N*^1^–CH_2_–*N*^2^-**5** isomer due to the steric effect. In the case of 1,3-dibromopropane two compounds were obtained due to the alkylation at *N*(2) to give 2-(3-bromopropyl)-triazole **8** and alkylation at *N*(1) was followed by cyclization at the indole nitrogen to form a condensed indolo-triazolo-diazepine **10**. Upon alkylation of 3-benzylsufanyl-5-(1*H*-indolyl)-1,2,4-triazole with di(bromomethyl)quinoxaline, two bis(triazolyl-methyl)quinoxaline isomers were separated and characterized as (–*N*^1^–CH_2_–*N*^1^–) **11** and (–*N*^2^–CH_2_–*N*^2^–) **12**. Single-crystal X-ray diffraction assisted the elucidation and confirmation of the structures of the isomers. An AM1 theoretical study explained the regioselectivity of the alkylation.

**Conclusions:**

On reacting *S*-protected 1,2,4-triazoles with various alkylating agents, only *N*(1) and *N*(2) attack the electrophilic carbons. *N*(2) alkylated isomers are preferentially formed.

**Electronic supplementary material:**

The online version of this article (doi:10.1186/s13065-016-0165-0) contains supplementary material, which is available to authorized users.

**Graphical abstract:**
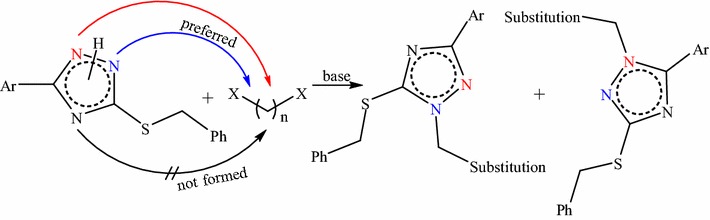
.

## Background

**Fig. 1:**
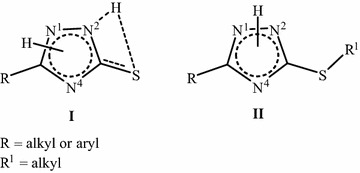
Structure of dihydro-1,2,4-triazole-thione **I** and *S*-protected 1,2,4-triazole **II**

After designing and applying a synthetic approach, structure elucidation and confirmation constitutes the second important step. Sometimes, spectroscopic techniques are not enough for structure confirmation, especially when the starting scaffolds have more than one site accessible to the reaction such as dihydro-1,2,4-triazole-thione **I** and *S*-protected 1,2,4-triazole **II** (Fig. 1). Here, the difficulty arises in assigning which nitrogen atom will be alkylated.

Most of published literature concentrated their studies on preparation of the parent triazoles **I** and the corresponding *S*-substituted analogs **II**. The reasons for this may be the high yields of the *S*-derivatives, and lack of interest to assign which nitrogen had reacted due to the extra effort needed for separation and structure determination. Nevertheless, the triazoles of type **I** and **II** were subjected to many biological investigations and showed a wide range of activities such as antimicrobial [1–3], antituberculosis [4], antiviral [5, 6], anti-inflammatory [7, 8], anticonvulsant [9] and antiproliferative [10] properties. Moreover, selective inhibition activities for COX-2 [11], urease [12] and trans-cinnamate 4-hydroxylase [13] were found. Some structures of the alkylsulfanyl-1,2,4-triazoles **II** were confirmed by single-crystal X-ray diffraction [14–16].

Structural reports dealing with the alkylation at nitrogen atoms of *S*-protected 1,2,4-triazoles with mono alkyl halides are very limited [17–19]. Despite the huge effort that has been done using NMR spectroscopy for determination of regioselectivity, conflicts in assignment of the alkylation site are found. For example, alkylation was proposed to include (*S*-, 2-*N*-) and (*S*-, 4-*N*-) in [17, 18], (*S*-, 2-*N*-) and (*S*-, 1-*N*-) was suggested in [3] but (*S*-, 1-*N*-) only was shown in [19]. Substitution at the sulfur followed by cyclization at nitrogen (*2*-*N*-) was proposed in [20, 21] whereas at nitrogen (*4*-*N*-) was found in [22]. Hence, the published assignments of the alkylation pattern in 1,2,4-triazoles seem unclear.

Hence, the present study is the first research work concerned with determination of the regioselectivity of alkylation upon reacting dihaloalkanes with an *S*-alkylated 1,2,4-triazole. Growing of single crystals of the products permitted elucidation of the alkylation site and supported the structure assignment of the remaining isomers by NMR.

## Results and discussion

**Scheme 1:**
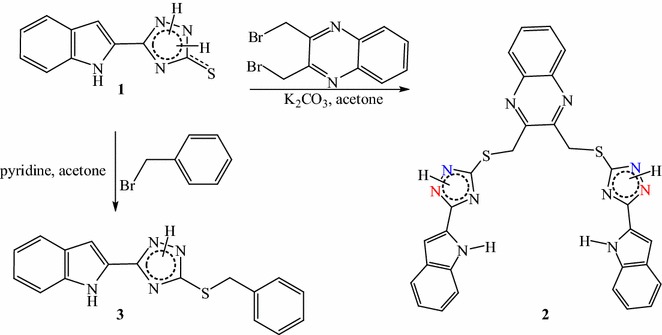
*S*-alkylation of 1,2,4-triazolethione **1**

Dihydro-1,2,4-triazolinethione **1** was coupled with (dibromomethyl)quinoxaline in the presence of K_2_CO_3_ and benzyl bromide using pyridine to afford the required *S*-protected 1,2,4-triazoles **2** and **3**, respectively (Scheme 1).

**Scheme 2:**
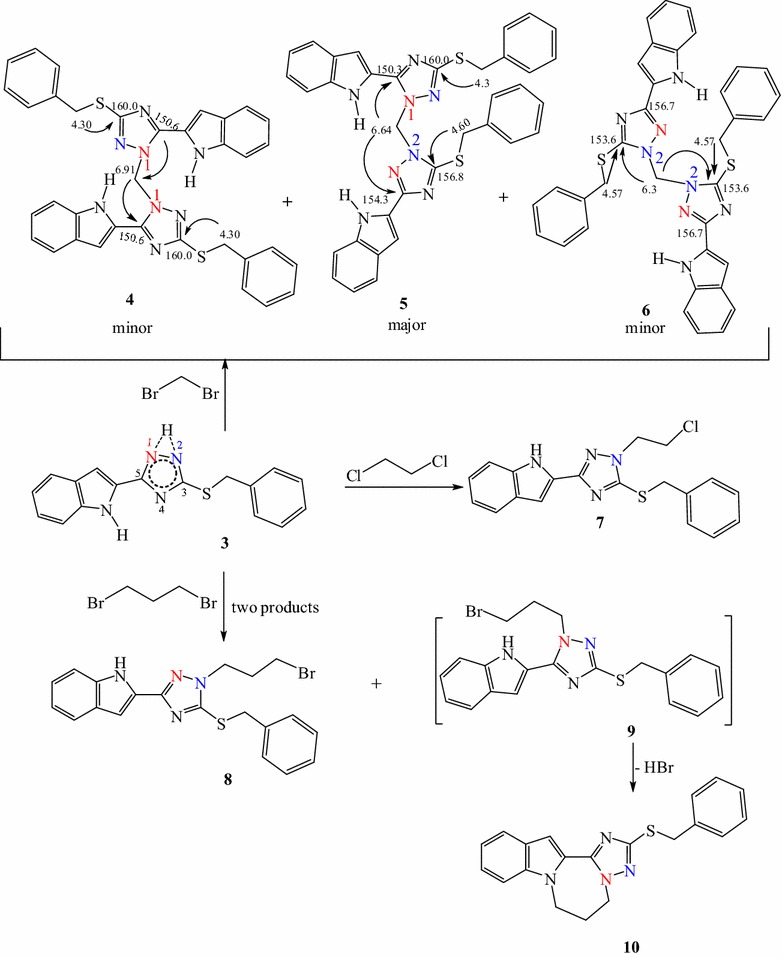
Products obtained from reaction of **3** with dibromomethane, 1,2-dichloroethane and 1,3-dibromopropane. HMBC schematic representation of different bis-(triazolyl)methane isomers

Dibromomethane as the shortest-chain alkylation reagent was first reacted with 3-benzylsulfanyl-5-(1*H*-indol-2-yl)-2*H*-1,2,4-triazole **3** in the presence of K_2_CO_3_ as an acid scavenger. Three isomers **4**–**6** were separated by column chromatography in 15, 50 and 10 % yields, respectively. Increasing the length of the carbon chain and reacting **3** with dichloroethane led to the replacement of one chlorine atom by the triazolyl moiety to give **7**. However, the use of 1,3-dibromopropane under the same conditions yielded **8** and **10** and the reaction was more selective towards **8** which formed in 60 % yield compared to **10** which formed in 28 % yield (Scheme 2).

**Scheme 3:**
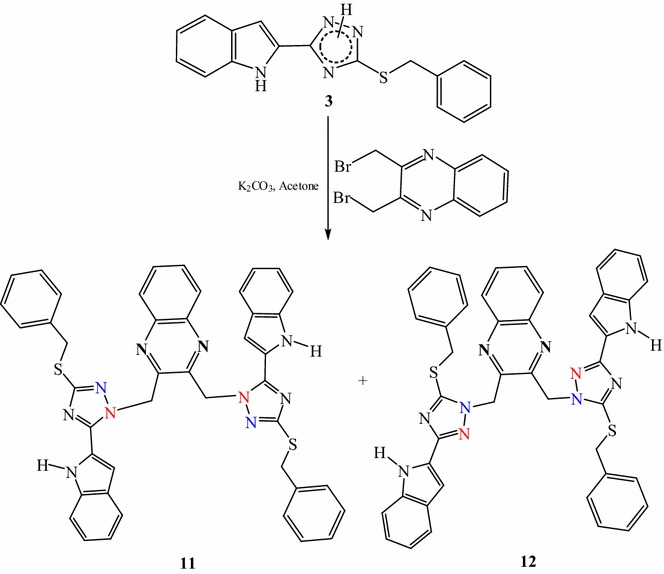
Reaction of *S*-benzyl-1,2,4-triazole with dibromomethylquinoxaline

Reaction of *S*-benzylated triazole **3** with (dibromomethyl)quinoxaline afforded two bis(triazolylmethyl) quinoxaline isomers **11** and **12** and no cyclization was observed (Scheme 3).

### Structure confirmation (Additional files 1 and 2)

The ^1^H NMR of **2** showed a singlet signal at 5.04 ppm for the two SCH_2_ groups and the protons of the indole and the quinoxaline appeared between 6.95 and 8.01 ppm. Two broad signals appeared at 11.70 and 14.43 ppm which are characteristic for indole and triazole NHs. ^13^C NMR showed the two benzyl methylene carbons (2 SCH_2_) at 36.16 ppm. The quinoxaline (CH) carbon signals appeared at 128.3 and 130.27 ppm, whereas its quaternary carbons appeared at 140.25 and 151.39 ppm. The ^1^H NMR of **3** shows two D_2_O exchangeable signals at 11.77 for the indole NH and 14.37 ppm for the triazole NH. The benzyl methylene protons appeared as singlet at 4.45 ppm and the corresponding methylene carbon appeared in ^13^C NMR at 35.66 ppm. The presence of a signal around 14.40 ppm in ^1^H NMR of **2** and **3** confirms that only one proton has been replaced. The absence of a thiocarbonyl (C = S) signal around 166.6 ppm [3] in the ^13^C NMR of **2** and **3** strongly recommends the alkylation of sulfur.

The NH signal at 14.37 ppm (in the spectrum of **3**) disappeared in the ^1^H NMR spectra of compounds **4**–**12** which means that this proton has been substituted. The ^1^H NMR of **4** showed the four methylene protons of the two benzyl groups at 4.30 ppm, the two protons of the methylene bridge connecting the two triazolyl moieties at 6.92 ppm and the indole NH proton at 12.01 ppm. The ^13^C NMR showed the two benzyl methylene carbons at 34.8 ppm while the methylene carbon connecting the two triazole moieties appeared at 60.0 ppm. The two triazole carbons (C-5_Triazol_, C-3_Triazol_) appeared at 150.60 and 160.04 ppm respectively.

The ^1^H NMR spectrum of **5** showed two signals for the four methylene protons of the two benzyl groups at 4.31 and 4.60 ppm while the two protons of the methylene bridge connecting the two triazole moieties appeared at 6.64 ppm. The NH protons of the two indole rings were found at 11.64 and 11.95 ppm. The ^13^C NMR displayed the two methylene carbons of the two benzyl groups at 34.8 and 37.4 ppm, whereas the methylene bridge carbon was found at 59.2 ppm. The four carbons of the two triazole rings were identified at 150.3, 154.3, 156.7 and 159.96, respectively.

**Fig. 2:**
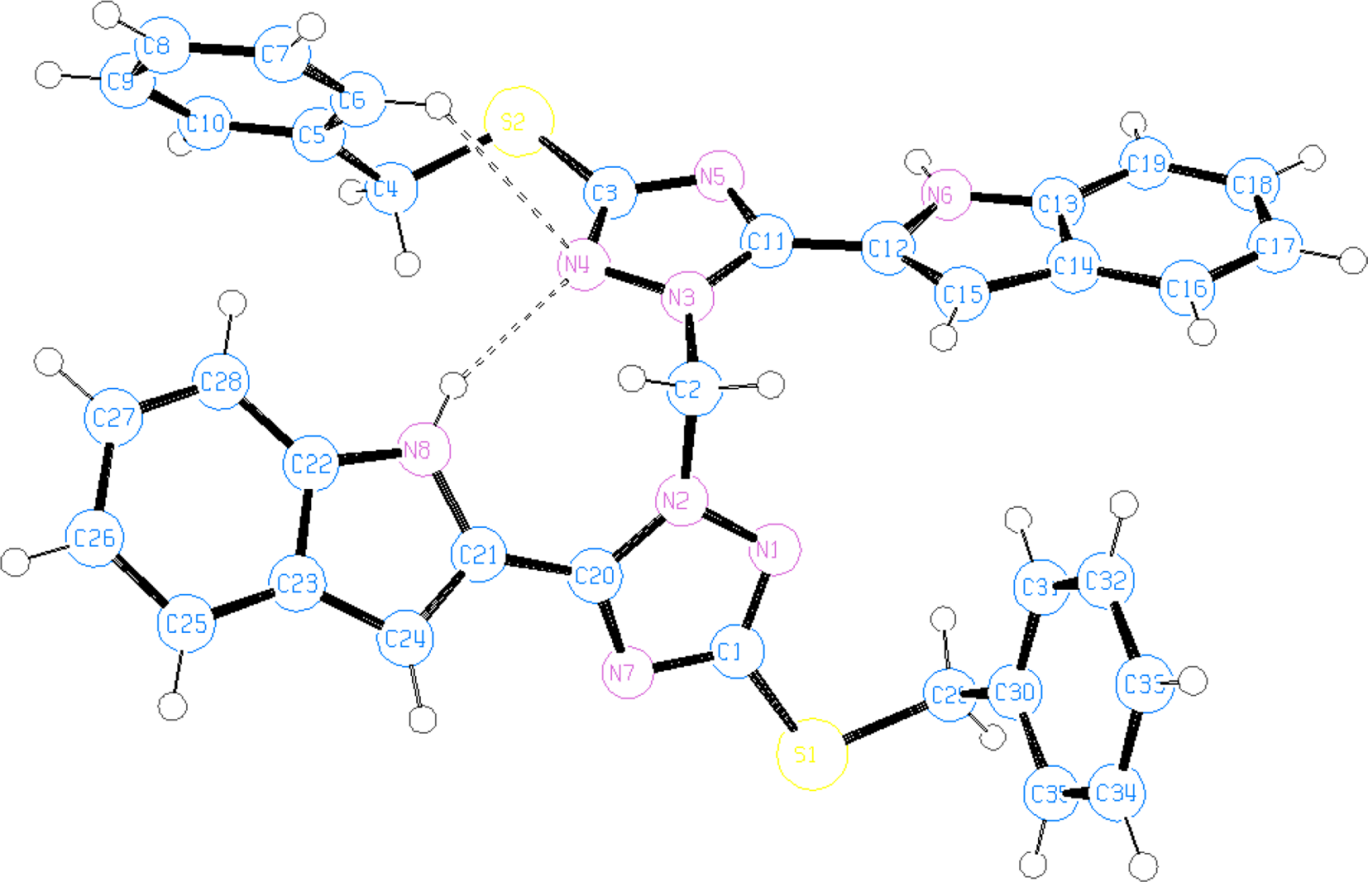
Ortep representation showing the *N*(2) attack in (bistriazolyl)methane **4** in addition to the effect of two intramolecular hydrogen bonds on the planarity of one indolyltriazole

**Fig. 3:**
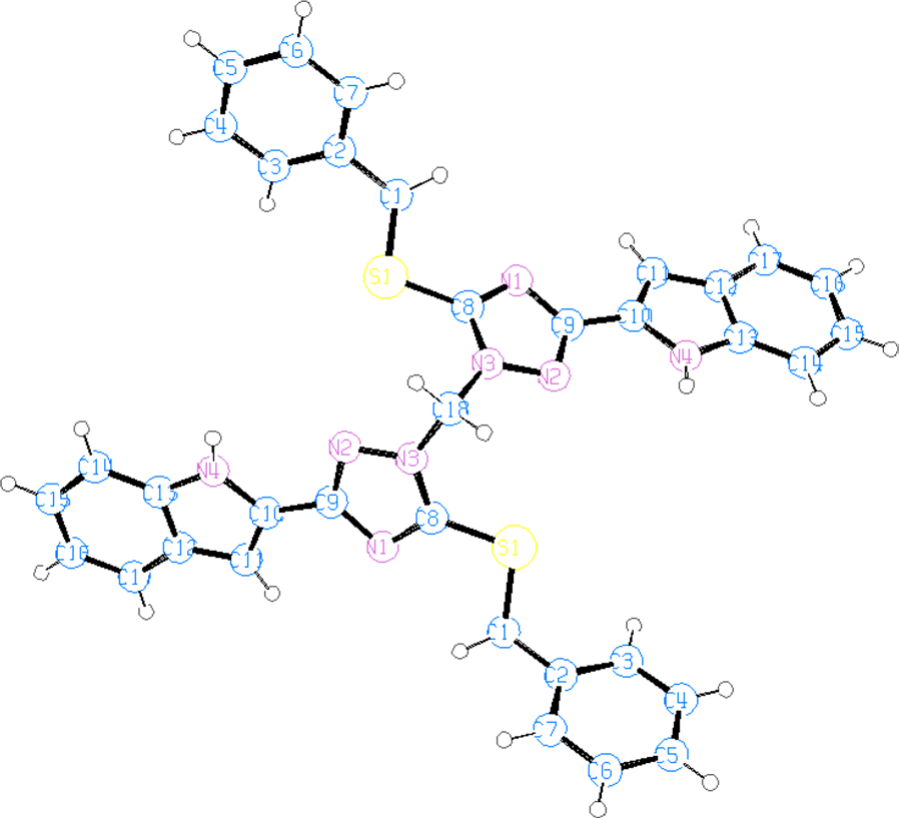
Ortep representation showing the *N*(1) attack in (bistriazolyl)methane **6**

The ^1^H NMR of **6** showed one signal of the benzyl methylene protons at 4.57 ppm and methylene carbon bridge (connecting the two triazole moieties) at 6.32 ppm. The NH of the two indole rings appeared at 11.61 ppm. The ^13^C NMR showed the two methylene carbons of the two benzyl groups at 37.5 ppm while the methylene carbon bridge appeared at 58.7 ppm. The triazole carbons are assigned to the signals at 153.6 (for C-3) and 156.7 ppm (for C-5). The above facts showed that in the case of **5** a distinct signal appeared for each benzyl methylene group assuming that this isomer is asymmetric in liquid NMR. However, the NMR of the isomers **4** and **6** showed only one signal representing the two benzyl methylene groups which suggests symmetric molecules. The HMBC schematic representation (Scheme 2) shows the coupling correlations of the methylene protons (benzyl and bridge) and the triazole carbons in the three isomers **4-6**. The HMBC spectrum of **4** showed a ^3^*J*_C,H_ coupling correlation between the benzyl methylene protons at 4.30 ppm and the triazole carbon at 160.0 ppm, while the connecting methylene protons at 6.92 ppm showed ^3^*J*_C,H_ coupling with the triazole carbon at 150.6 ppm. In the HMBC of isomer **6**, both benzyl methylene protons at 4.57 ppm and the connecting methylene protons at 6.32 ppm displayed a coupling correlations ^3^*J*_C,H_ to the same triazole carbon at 153.6 ppm. In the HMBC of the isomer **5** the situation is different, because there are four signals of the triazole carbons and two signals of the methylene groups of the two benzyls. As a result, the benzyl methylene protons at 4.31 ppm showed ^3^*J*_C,H_ coupling to the triazole carbon at 160.0 ppm, whereas the other benzyl methylene protons at 4.60 ppm displayed the correlation ^3^*J*_C,H_ to a triazole carbon at 156.8 ppm. The connecting methylene protons at 6.64 ppm showed a ^3^*J*_C,H_ correlation to the two triazole carbons at 150.3 and 154.3 ppm. The structures of **4** and **6** were further confirmed by single crystal X-ray diffraction analysis as shown in Figs. 2, 3.

The ^1^H NMR spectrum of **7** showed three methylene groups (-NCH_2_CH_2_Cl and -SCH_2_Ph) at 3.74, 4.25 and 4.44 ppm. The three related methylene carbons were detected in ^13^C NMR at 38.9, 41.4 and 49.8 ppm. The two triazole carbons appeared at 152.8 and 157.1 ppm. The EI mass spectrum showed the molecular ion peak at 368.0 (80.2) and 370.0 (31.9) for (M + 2)^+^, indicating the presence of chlorine.

The ^1^H NMR of **8** displayed four methylene group signals at 2.48, 3.74, 4.42 and 4.81 ppm. The ^13^C NMR showed the respective four methylene carbons at 31.0, 31.9, 37.4 and 46.6 ppm and the two triazole carbons at 151.4 and 156.2 ppm. The mass spectrum (ESI) showed two peaks at m/z 427 (98.2 %) for (M + H)^+^ and 429 (100 %) for (M + H+2)^+^ which strongly recommend the presence of the bromine atom.

**Fig. 4:**
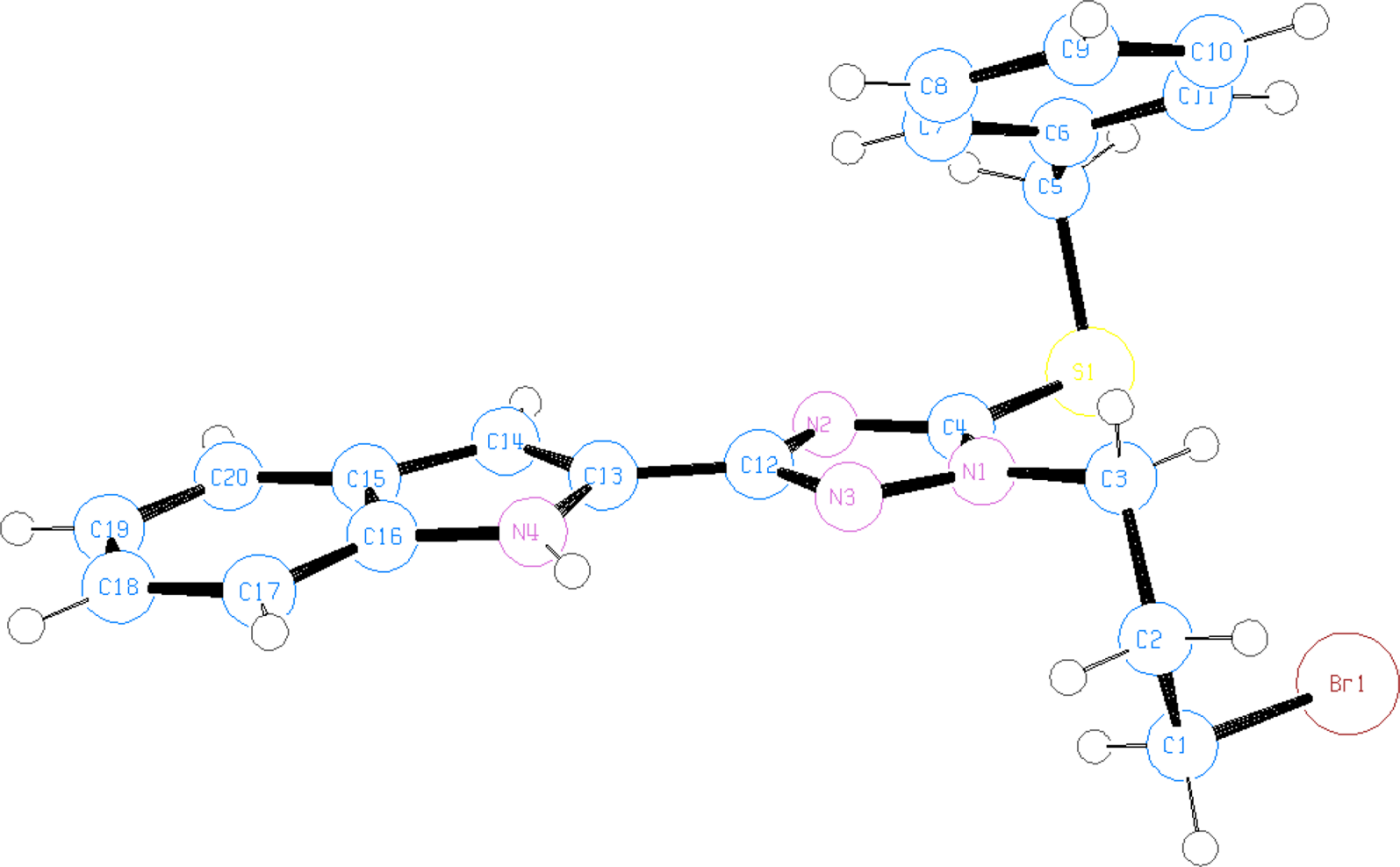
Ortep representation of **8** showing the attachment of 3-bromopropyl to *N*(2)

**Fig. 5:**
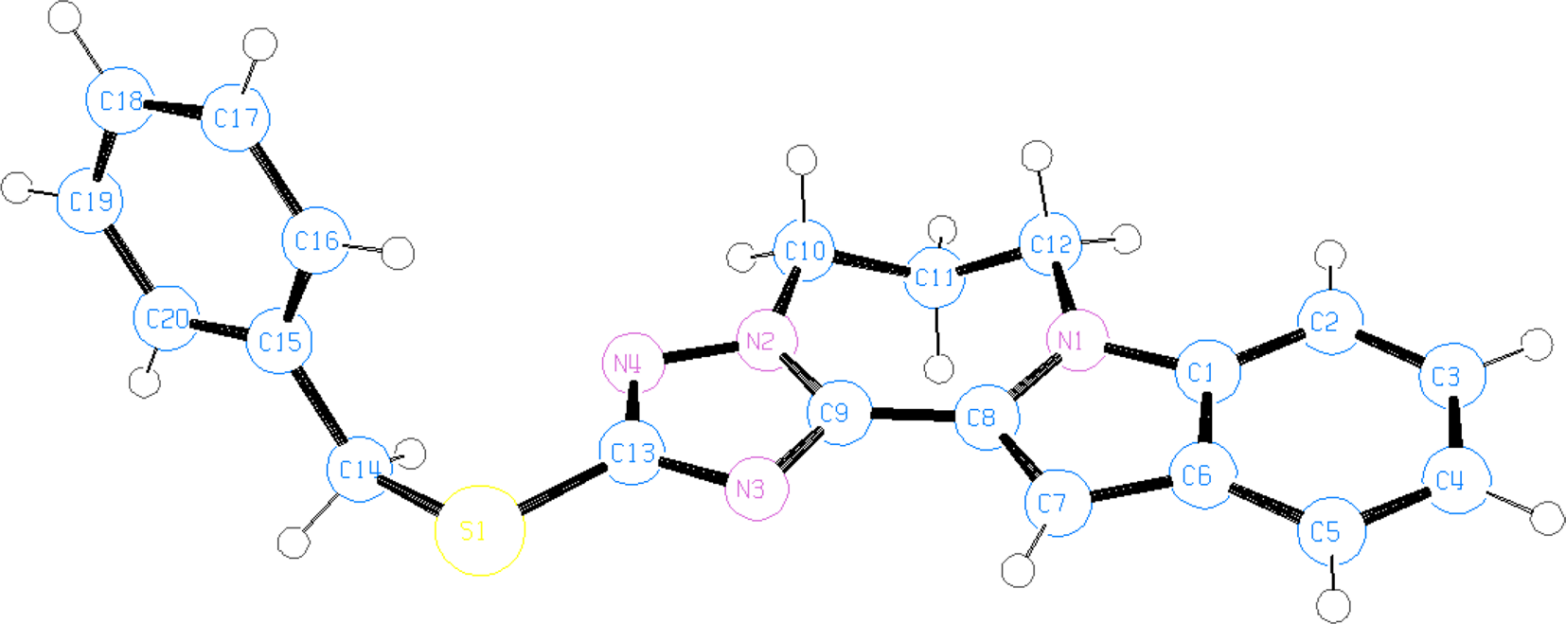
Ortep representation of indolo-triazolo-diazepine **10**

The spectra of **10** showed three signals at 2.43–2.48, 4.39, and 4.52–4.54 ppm for the four methylene groups whereas the related methylene carbons appeared at 25.2, 35.0, 44.7 and 51.7 ppm. The chemical shifts of 44.7 and 51.7 ppm indicate that there are two methylene carbons attached to two different nitrogen atoms (triazole and indole). The two triazole carbon signals were found at 148.6 and 158.5 ppm. This indicates that one bromine atom of 1,3-dibromopropane is removed first due to the attack of the triazole nitrogen and due to a proximity arrangement to the indole ring, the second bromine atom is then lost upon the attachment of the methylene carbon to the nitrogen of the indole. The structures of **8** and **10** were further confirmed by X-ray single crystal structures (Figs. 4, 5, Additional file 1).

The ^1^H NMR spectrum of **11** displayed the methylene protons of the benzyl group as singlet at 4.34 ppm while the methylene protons attaching triazole and quinoxaline rings appeared as a singlet at 6.14 ppm. The respective methylene carbons appeared at 36.44 and 51.97 ppm. All aromatic protons appeared between 6.90 and 8.20 ppm and the indole NH appeared at 10.56 ppm. The triazole carbons appeared at 149.05 and 160.33 ppm.

The ^1^H NMR spectrum of **12** showed the indole NH signal at 11.60 ppm. The methylene protons of the benzyl group were found at 5.22 ppm while the methylene protons linking triazole and quinoxaline rings appeared at 5.42 ppm. The respective methylene carbons were found at 37.9 and 52.3 ppm, respectively. Moreover, the two carbon signals of the triazole carbons appeared at 152.4 and 156.8 ppm.

### X-ray analysis

Single-crystal X-ray diffraction analysis afforded unambiguous structural assignments of the isomers formed. All single crystals were grown by slow evaporation of the eluent (ethyl acetate/hexane 2:8). Crystal data showed that crystals of **4** and **6** have the same empirical formula and formula weight. Compound **6** crystallizes in the *C*2/*c* space group with *Z* = 4, which means that the molecules of **6** occupy special positions. Atom C18 is located at a twofold proper rotation axis. This means that in the case of **6** the molecular conformation in liquid (NMR) and in the solid state is the same. On the other hand, in the case of **4** the space group is *P*2_1_/*c*, *Z* = 4, which means that the molecules occupy general positions and hence can be asymmetric. In liquid (NMR) they appear symmetric which indicates that the molecular conformation is a soft molecular parameter and the crystal lattice arrangement changes it. The crystallographic data of the isomers **4** and **6** are shown in Table 1.

**Table 1:** The crystal data, details on data collection and refinement of **4** and **6**

Compound	**4**	**6**
Formula	C_35_H_28_N_8_S_2_	C_35_H_28_N_8_S_2_
*D*_*calc.*_/g cm^−3^	1.414	1.416
*µ*/mm^−1^	1.975	0.224
Formula weight	624.77	624.77
Colour	Colourless	Colourless
Shape	Plate	Rod
Max size/mm	0.31	0.35
Mid size/mm	0.18	0.05
Min size/mm	0.02	0.04
*T*/K	123.0	123.0
Crystal system	Monoclinic	Monoclinic
Space group	P2_1_/c	C2/c
*a*/Å	14.8390 (2)	32.9005 (5)
*b*/Å	11.33640 (10)	8.04840 (12)
*c*/Å	17.8545 (2)	11.51861 (19)
*α*/^°^	90	90
*β*/^°^	102.2920 (10)	106.0192 (17)
*γ*/^°^	90	90
V/Å^3^	2934.64 (6)	2931.65 (8)
*Z*	4	4
*Z’*	1	0.5
Ɵ_*min*_/^°^	4.652	2.576
Ɵ_*max*_/^°^	73.662	26.241
Measured Refl.	23347	10434
Independent Refl.	5819	2927
Reflections used	5274	2824
*R*_*int*_	0.0245	0.0187
Parameters	406	210
Restraints	36	0
Largest peak	0.871	0.285
Deepest hole	−0.460	−0.194
GooF	1.049	1.033
*wR*_*2*_ (all data)	0.1058	0.0852
*wR*_*2*_	0.1015	0.0841
*R*_*1*_ (all data)	0.0428	0.0328
*R*_*1*_	0.0388	0.0317
CCDC	1,420,937	1,420,938

2-(3-Bromopropyl)-triazole **8** crystallized in the monoclinic space group P21/c. The indolo-triazolo-diazepine **10** crystallized as triclinic space group P-1. The crystallographic data of compounds **8** and **10** are given in Table 2.

**Table 2:** The crystal data, details on data collection and refinement of **8** and **10**

Compound	**8**	**10**
Formula	C_20_H_19_N_4_SBr	C_20_H_18_N_4_S
*D*_*calc.*_/g cm^−3^	1.510	1.406
*µ*/mm^−1^	4.100	1.826
Formula weight	427.36	346.44
Colour	Translucent colourless	Colourless
Shape	Rod	Stick
Max size/mm	0.52	0.51
Mid size/mm	0.10	0.14
Min size/mm	0.04	0.07
*T*/K	123	123.0
Crystal system	Monoclinic	Triclinic
Space group	P2_1_/c	P-1
*a*/Å	9.13384 (13)	9.0486 (4)
*b*/Å	21.7680 (3)	9.1081 (5)
*c*/Å	9.47711 (13)	11.7993 (6)
*α*/^°^	90	68.513 (5)
*β*/^°^	93.8246 (13)	80.348 (4)
*γ*/^°^	90	64.771 (5)
V/Å^3^	1880.09 (5)	818.47 (8)
*Z*	4	2
*Z’*	1	1
Ɵ_*min*_/^°^	4.062	4.027
Ɵ_*max*_/^°^	71.438	73.118
Measured Refl.	6987	5423
Independent Refl.	3533	3154
Reflections used	3024	2966
*R*_*int*_	0.0220	0.0149
Parameters	238	226
Restraints	0	0
Largest peak	0.350	0.278
Deepest hole	−0.464	−0.279
GooF	1.072	1.046
*wR*_*2*_ (all data)	0.0802	0.0840
*wR*_*2*_	0.0776	0.0822
*R*_*1*_ (all data)	0.0356	0.0340
*R*_*1*_	0.0293	0.0320
CCDC	1420939	1420940

In General, all crystalline compounds **4**, **6**, **8** and **10** are nonplanar. This is because the benzyl groups are almost perpendicularly positioned to the plane of indolyltriazole system (Tables 3, 4). In crystals of **6**, **8** and **10** the indole and triazole rings are located nearly in the same plane (torsion angles are around 10°), except in one half of isomer **4** in which the two intramolecular hydrogen bonds N(8)–H(8 N)^**…**^N(4) and C(6)–H(6a)^**…**^N(4) appeared in the mono-structure (Fig. 1) twisted the indole ring and deviated this planarity by making torsion angles N(7)–C(20)–C(21)–N(8) of 148.37° and at N(7)–C(20)–C(21)–C(24) of 31.1°, respectively (Tables 3).

**Table 3:**
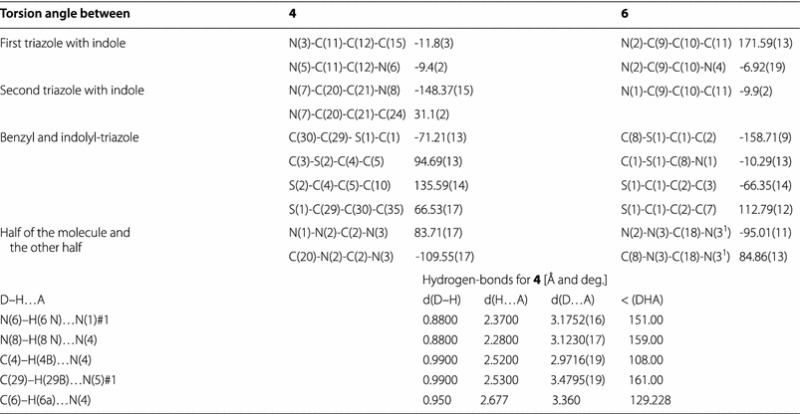
Selected torsion angles and hydrogen bonding in crystals of **4** and **6**

**Table 4:**
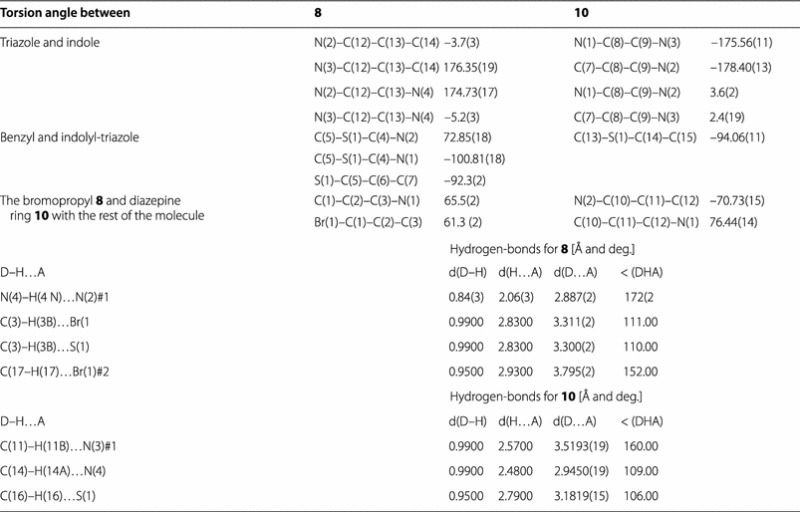
Selected torsion angles and hydrogen bonding in crystals of **8** and **10**

In crystal **10**, the diazepine ring is not planar because torsion angles at N(2)–C(10)–C(11)–C(12) of torsion angles at and at C(10)–C(11)–C(12)–N(1) of 76.44° appeared.

**Fig. 6:**
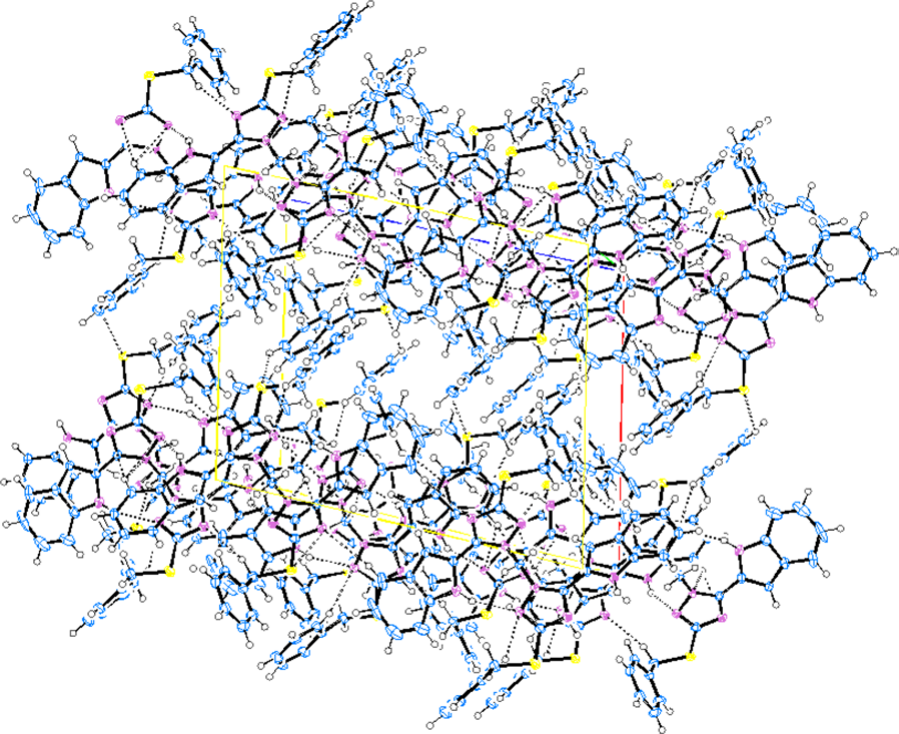
Packing diagram of **4**. *Dashed lines* represent hydrogen bonds

**Fig. 7:**
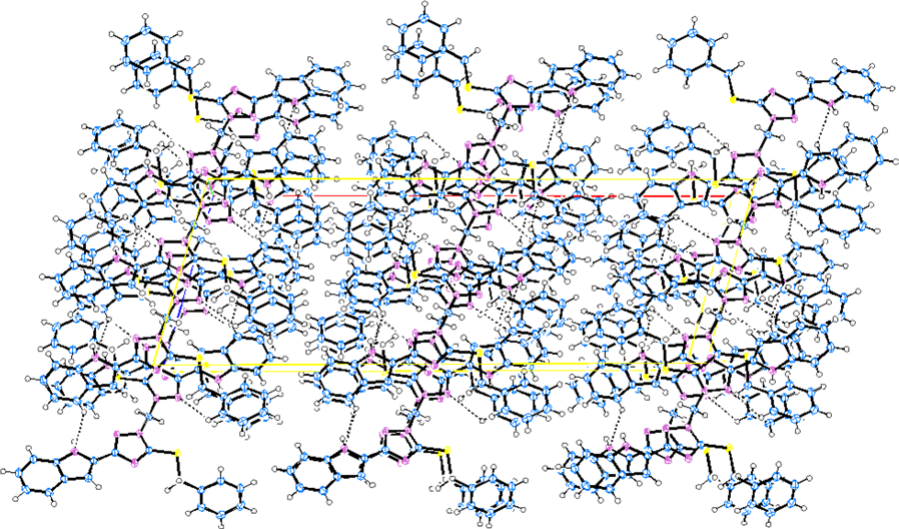
Packing of the molecules in the unit cell of **6**. *Dashed lines* represent hydrogen bonds

**Fig. 8:**
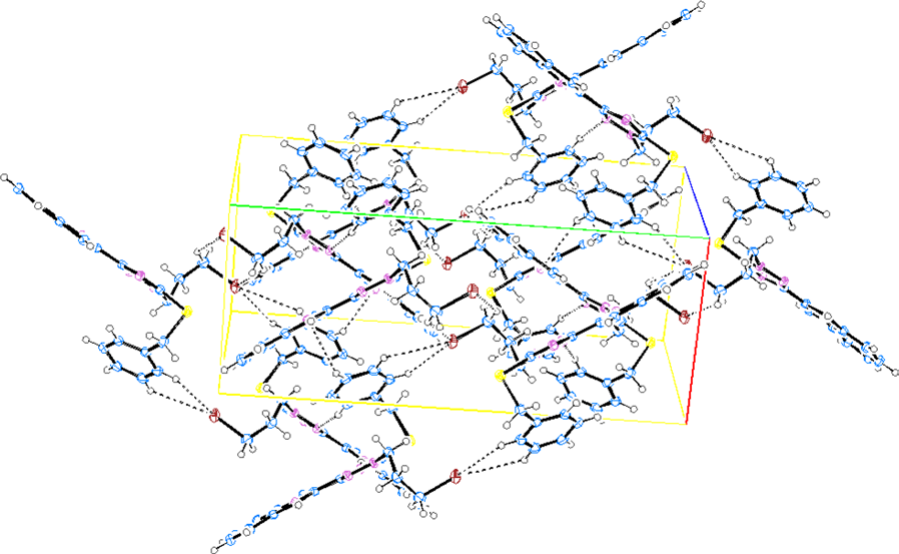
Packing diagram of **8**. *Dashed lines* represent hydrogen bonds

**Fig. 9:**
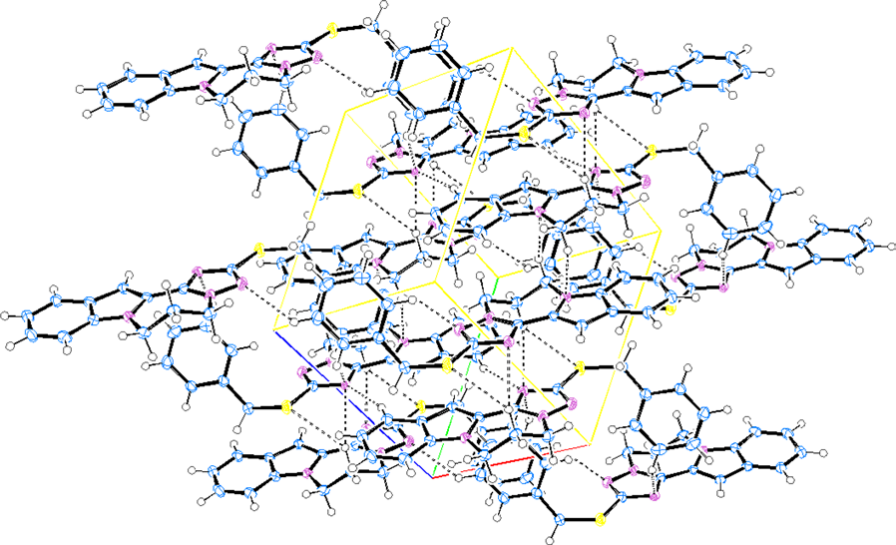
Packing diagram of **10**. *Dashed lines* represent hydrogen bonds

The supramolecular structures are stabilized in the 3D network by the intermolecular hydrogen bonding and van der Waals interactions (Figs. 6, 7, 8, 9). Both, **8** and **10** are centrosymmetric and no isomers are present due to the absence of a stereo center.

### AM1 computational analysis of bis(triazolyl)methane isomers

The calculated empirical data of the three isomers **4**–**6** shows that isomer **5** has the lowest steric energy 16.2704 kcal/mol. This suggests that it will be formed with the highest yield with respect to the remaining two isomers **4** and **6**. This is in well agreement with the yields found in the synthesis of **5**, **4**, and **6** (being 50, 15 and 10 %, respectively). Moreover, we found that the charge density on the triazole nitrogens is mainly delocalized on *N*(1) and *N*(2). Accordingly, if the negative charge is located on *N*(1) or *N*(2), they will be preferentially attack the electrophilic carbon (Table 5) in alkylation reactions.

**Table 5:** AM1 calculations of triazole nitrogens, heat of formation and steric energy in **4-6**

	**4**		**5**		**6**	
Charges (Mulliken charges):	N(10)	-0.0998	N(10)	-0.0467	*N(10)*	-*0.1631*
	*N(11)*	-*0.1586*	*N(11)*	-*0.1704*	N(11)	-0.0603
	C(12)	0.0315	C(12)	0.0401	C(12)	-0.0302
	N(13)	-0.1167	N(13)	-0.1102	N(13)	-0.1686
	C(14)	-0.2673	C(14)	-0.2920	C(14)	-0.1775
	S(15)	0.3333	S(15)	0.2792	S(15)	0.3004
	N(27)	-0.1106	*N(27)*	-*0.1577*	*N(27)*	-*0.1633*
	*N(28)*	-*0.1686*	N(28)	-0.0500	N(28)	-0.0599
	0.0487	C(29)	C(29)	-0.0363	C(29)	-0.0302
	N(30)	-0.1025	N(30)	-0.1138	N(30)	-0.1690
	C(31)	-0.2605	C(31)	-0.1921	C(31)	-0.1773
	S(32)	0.3425	S(32)	0.2906	S(32)	0.3002
Heat of formation (Kcal/Mol)	359.6466		356.9822		349.1808	
Steric energy (Kcal/Mol)	37.7514		16.2704		85.3608	

## Conclusion

Upon alkylation of *S*-substituted 1,2,4-triazoles with dihaloalkanes, products will appear preferentially resulting from the attack of the alkyl moiety at *N*(1) or *N*(2) of the triazole. Theoretical calculations reveal that there is no chance to obtain an alkylation on *N*(4) due to the steric energy. This is also concluded from HMBC and ^13^C NMR which additionally help in the structure assignment of the positions of the two carbon atoms of the alkylated triazole. If they appear around 152.0 and 157.0 ppm substitution at the nitrogen atom nearest to sulfur, i.e. *N*(2), can be deduced. However, a substitution on the nitrogen atom far from sulfur, i.e. *N*(1), can be derived if the carbon atoms of the alkylated triazole appear around 149.0 ppm and 160.0 ppm. These results were confirmed by single-crystal X-ray diffraction.

## Experimental

Melting points were determined with a melting-temperature apparatus (SMP10) in open capillaries and are uncorrected. The progress of all reactions was monitored by TLC pre-coated plates with silica gel 60 F_254_ of a thickness of 0.25 mm (Merck). Detection was achieved by UV light absorption and/or treatment with a solution of 10 % H_2_SO_4_ in aqueous methanol with subsequent heating. For flash chromatography commercial silica gel 60 was used with particle sizes 0.040–0.063 mm (230–400 mesh ASTM). Solvents were purified by simple distillation. Nuclear magnetic resonance (^1^H NMR, ^13^C NMR and 2D NMR) spectra were determined in CDCl_3_ and DMSO-*d*_6_ and were recorded on Bruker AC 300–600 spectrometers, with tetramethylsilane as internal standard. Chemical shifts are reported in δ (ppm) and coupling constants are given in Hz. The assignment of exchangeable O*H* and N*H* was confirmed by addition of D_2_O. EI mass spectra were recorded with a Finnigan MAT312 and a Jeol JMS.600H mass spectrometer. HREI mass spectral data were recorded with a Finnigan MAT 95XP instrument. FABMS was recorded with the Jeol JMS HX110 mass spectrometer. ESI were recorded with an Applied Biosystems QStar XL instrument. The crystallographic measurements were performed on an Agilent (formerly Oxford diffraction) SuperNova Atlas CCD diffractometer. The structures were solved by direct methods (*SIR97*) and refined by full-matrix anisotropic least squares (SHELXL-2014/7).

### Synthesis of *S*-alkylated derivatives (2, 3)

To a mixture of traizole **1** (1.0 mmol) in acetone (10 ml) and appropriate base (4.0 mmol), 2,3-bis(bromomethyl)quinoxaline (0.5 mmol) or benzyl bromide (1.1 mmol) were added and stirring was continued overnight. The reaction mixture was filtered. The solvent was evaporated in *vacuo* and cold water was added. The solids formed were collected by filtration, dried, and separated by column chromatography (ethyl acetate/hexane 1:1) **2** or crystallization from ethanol **3**.

#### 2,3-Bis((5-(1*H*-indol-2-yl)-2*H*-1,2,4-triazol-3-ylsulfanyl)methyl)quinoxaline (2)

White solid, Yield 71 %; m.p. 203_decomp_.  °C; R_*f*_ 0.35 (ethyl acetate/n-hexane 6:4); ^1^H NMR (DMSO-*d*_6_, 300 MHz) δ 5.04 (s, 4 H, 2 CH_2_), 6.95 (s, 2 H, 2 H-3_Indol_), 7.00 (dd, 2 H, *J*_4,5_ ≈ *J*_5,6_ 7.5, Hz, 2 H-5_Indol_), 7.13 (dd, 2 H, *J*_5,6_ 7.5, *J*_6,7_ 8.1 Hz, 2 H-6_Indol_), 7.41 (d, 2 H, *J*_6,7_ 8.1 Hz, 2 H-7_Indol_), 7.56 (d, 2 H, *J*_4,5_ 7.5 Hz, 2 H-4_Indol_), 7.78–7.82 (m, 2 H, 2 CH_quinoxalin_), 7.97–8.01 (m, 2 H, 2 CH_quinoxalin_), 11.70 (br. s, 2 H, NH_Indol_), 14.43 (br. s, 2 H, NH_Triazol_); ^13^C NMR (DMSO-*d*_6_, 100 MHz) δ 36.16 (2 CH_2_), 101.70 (2 C-3_Indol_), 111.99 (2 C-7_Indol_), 119.72 (2 C-5_Indol_), 120.74 (2 C-4_Indol_), 122.70 (2 C-2_Indol_, 2 C-6_Indol_), 127.2 (2 C-3a_Indol_), 128.30 (2 CH_quinoxalin_), 130.27 (2 CH_quinoxalin_), 136.91 (2 C-7a_Indol_, 2 C-5_Triazol_, 2 C-3_Triazol_), 140.25 (2 C_quinoxalin_), 151.39 (2 C_quinoxalin_); HRMS (FAB +ve) calcd for C_30_H_23_N_10_S_2_ M + H)^+^: 587.15486. Found: 587.1508.

#### 3-Benzylsulfanyl-5-(1*H*-indol-2-yl)-2*H*-1,2,4-triazole (3)

Yield 97 %; m.p. 219–220 °C; R_*f*_ 0.49 (ethyl acetate/n-hexane 4:6); ^1^H NMR (DMSO-*d*_6_, 300 MHz) δ 4.45 (s, 2 H, C*H*_2_Ph), 6.99–7.05 (m, 2H, H-3_Indol_, H-5_Indol_), 7.15 (dd, 1H, *J*_5,6_ 7.3, *J*_6,7_ 7.7 Hz, H-6_Indol_), 7.21–7.45 (m, 6 H, H-7_Indol_, 5H_Ph_), 7.59 (d, 1 H, *J*_4,5_ 7.9 Hz, H-4_Indol_), 11.77 (br. s, 1H, NH_Indol_), 14.37 (br. s, H, NH_Triazol_); ^13^C NMR (DMSO-*d*_6_, 75 MHz) δ 35.66 (*C*H_2_Ph), 100.99 (C-3_Indol_), 111.94 (C-7_Indol_), 119.67 (C-5_Indol_), 120.66, 122.60, 127.27, 127.67 (C-2_Indol_, C-4_Indol_, C-6_Indol_, CH_Ph_, C-3a_Indol_), 128.42 (2 CH_Ph_), 128.83 (2 CH_Ph_), 136.84, 137.64 (C-7a_Indol_, C-3_Triazol_, C-5_Triazol_, C_Ph_); HRMS (EI) calcd for C_17_H_14_N_4_S (M^+.^): 306.0939. Found: 306.0886.

#### Alkylation of *S*-benzylated triazole 3

To a mixture of traizole **3** (1.0 mmol) in acetone (10 ml) and K_2_CO_3_ (2.1 mmol), appropriate dihaloalkane compound (0.5 mmol) was added and stirring was continued overnight. The reaction mixture was filtered and dried. Then, the products were separated by column chromatography (ethyl acetate/hexane 0.5:9.5).

#### Bis(3-benzylsulfanyl-5-(1*H*-indol-2-yl)-1*H*-1,2,4-triazol-1-yl)methane (4)

Colorless needle crystals; Yield 15 %; m.p. 218–219 °C; R_*f*_ 0.73 (ethyl acetate/n-hexane 4:6); ^1^H NMR (DMSO-*d*_6_, 300 MHz) δ 4.30 (s, 4H, 2 SCH_2Ph_), 6.92 (s, 2H, CH_2_), 7.08 (dd, 2H, *J*_4,5_ = 7.9, *J*_5,6_ = 7.3 Hz, H-5_Indol_), 7.16–7.30 (m, 12H, H-6_Indol_, 10 H_Ph_), 7.48–7.51 (m, 4H, H-3_Indol_, H-7_Indol_), 7.62–7.65 (d, 1H, *J*_4,5_ = 7.9 Hz, H-4_Indol_), 12.01 (br. s, 2H, NH_Indol_); ^13^C NMR (DMSO-*d*_6_, 75 MHz) δ 34.8 (2 SCH_2_Ph), 60.0 (CH_2_), 104.8 (C-3_Indol_), 112.2 (C-7_Indol_), 120.1 (C-5_Indol_), 121.3 (C-4_Indol_), 123.1 (C-2_Indol_), 123.7 (C-6_Indol_), 127.2 (CH_Ph_), 127.6 (C-3a_Indol_, C_Ph_), 128.3 (2 CH_Ph_), 128.9 (2 CH_Ph_), 137.1, 137.7 (C-7a_Indol_, C_Ph_), 150.6 (C-5_Triazol_), 160.04 (C-3_Triazol_); HRMS (ESI) calcd for C_35_H_29_N_8_S_2_ (M + H)^+^: 625.1951. Found: 625.1900.

#### 1,2-Bis(3-benzylsulfanyl-5-(1*H*-indol-2-yl)-1*H*-1,2,4-triazol-1-yl)methane (5)

Colorless crystals; Yield 50 %; m.p. 214-215 °C; R_*f*_ 0.71 (ethyl acetate/n-hexane 4:6); ^1^H NMR (DMSO-*d*_6_, 300 MHz) δ 4.31 (s, 2H, CH_2_), 4.60 (s, 2H, CH_2_), 6.64 (s, 2H, CH_2_), 6.98–7.03 (m, 2H, H-3_Indol_, H-5_Indol_), 7.09–7.19 (m, 5H, H-6_Indol_, H-5́_Indol_, 3H_Ph_), 7.23–7.33 (m, 6H, H-6́_Indol_, 5H_Ph_), 7.40–7.51 (m, 5H, H-3́_Indol_, H-7_Indol_, H-7́_Indol_, 2H_Ph_), 7.57 (d, 1H, *J*_4,5_ = 7.8 Hz, H-4_Indol_), 7.73 (d, 1H, *J*_4́,5́_ = 7.9 Hz, H-4́_Indol_), 11.65 (br. s, 1H, NH_Indol_), 11.95 (br. s, 1H, NH́_Indol_); ^13^C NMR (DMSO-*d*_6_, 75 MHz) δ 34.8 (SCH_2_Ph), 37.4 (SCH_2_Ph), 59.2 (CH_2_), 101.5 (C-3_Indol_), 104.9 (C-3́_Indol_), 111.9, 112.1 (C-7_Indol_, C-7́_Indol_), 119.6 (C-5_Indol_), 120.1, 120.6, 121.4, 122.5, 123.1 (C-4_Indol_, C-4́_Indol_, C-5́_Indol_, C-6_Indol_, C-6́_Indol_), (C-2_Indol_,), 127.1, 127.6 (C_Ph_, Ć_Ph_), 127.7, 128.2, 128.3, 128.5, 128.9, 130 (C-3a_Indol_, 2 CH_Ph_, C-3á_Indol_, 2 CH_Ph_, 2 CH_Ph́_, 2 CH_Ph́_), 136.9, 137.0, 137.1, 137.7 (C-7a_Indol_, C-7á_Indol_, C_Ph_, C_Ph́_), 150.3, 154.3, 156.7, 159.96 (C-3_Triazol_, C-3ʹ_Triazol_, C-5_Triazol_, C-5́_Triazol_); HRMS (EI) calcd for C_35_H_28_N_8_S_2_ (M^+.^): 624.1878. Found: 624.1867.

#### Bis(3-benzylsulfanyl-5-(1*H*-indol-2-yl)-1*H*-1,2,4-triazol-2-yl)methane (6)

Colorless sunny crystals; Yield 10 %; m.p. 249–250 °C; R_*f*_ 0.65 (ethyl acetate/n-hexane 4:6); ^1^H NMR (DMSO-*d*_6_, 300 MHz) δ 4.57 (s, 4H, 2 SCH_2Ph_), 6.32 (s, 2H, CH_2_), 6.94 (s, 2H, 2H-3_Indol_), 7.18 (dd, 2H, *J*_4,5_ = 7.8, *J*_5,6_ = 7.5 Hz, 2H-5_Indol_), 7.12 (dd, 2H, *J*_5,6_ = 7.5, *J*_6,7_ > 8.0 Hz, 2H-6_Indol_), 7.23–7.32 (m, 6H, 6H_ph_), 7.41–7.46 (m, 6H, 2H-7_Indol_, 4H_Ph_), 7.56 (d, 1H, *J*_4,5_ = 7.8 Hz, 2H-4_Indol_), 11.61 (br. s, 2H, NH_Indol_); ^13^C NMR (DMSO-*d*_6_, 100 MHz) δ 37.5 (2 S*C*H_2_Ph), 58.7 (CH_2_), 101.4 (2C-3_Indol_), 112.0 (2C-7_Indol_), 119.6 (2C-5_Indol_), 120.6 (2C-2_Indol_, 2C-4_Indol_), 122.5 (C-6_Indol_), 127.65 (2CH_Ph_), 127.7, 128.3 (2C-3a_Indol_, 2C_Ph_), 128.5 (4 CH_Ph_), 129.08 (4 CH_Ph_), 136.75, 137.0 (2C-7a_Indol_, 2C_Ph_), 153.6 (2C-3_Triazol_), 156.7 (2C-5_Triazol_); HRMS (ESI) calcd for C_35_H_29_N_8_S_2_ (M + H)^+^: 625.1951. Found: 625.1900.

#### 3-Benzylsulfanyl-2-chloroethyl-5-(1*H*-indol-2-yl)-1*H*-1,2,4-triazole (7)

Colorless crystals, Yield 55 %; m.p. 126–127 °C; R_*f*_ 0.80 (ethyl acetate/n-hexane 6:4); ^1^H NMR (CDCL_3_, 400 MHz) δ 3.74 (t, 2 H, *J* 6.4 Hz, CH_2_Cl), 4.25 (t, 2 H, *J* 6.4 Hz, NCH_2_), 4.44 (s, 2 H, SCH_2_Ph), 7.10–7.14 (m, 2 H, H-3_Indol_, H-5_Indol_), 7.23 (dd, 1 H, *J*_5,6_ 6.8, *J*_6,7_ 8.4 Hz, H-6_Indol_), 7.27–7.29 (m, 5 H, Ph), 7.39 (d, 1 H, *J*_6,7_ 8.0 Hz, H-7_Indol_), 7.67 (d, 1 H, *J*_4,5_ 8.0 Hz, H-4_Indol_), 9.06 (br. s, 1H, NH_Indol_); ^13^C NMR (CDCl_3_, 100 MHz) δ 38.95 (SCH_2_Ph), 41.38 (CH_2_Cl), 49.81 (NCH_2_), 101.90 (C-3_Indol_), 111.18 (C-7_Indol_), 120.31 (C-5_Indol_), 121.34 (C-4_Indol_), 123.21 (C-2_Indol_, C-6_Indol_), 127.94 (CH_Ph_), 128.68 (C-3a_Indol_), 128.80, 128.93 (4 CH_Ph_), 136.36, 136.56 (C-7a_Indol_, C_Ph_), 152.76 (C-5_Triazol_), 157.07 (C-2_Triazol_); LRMS-EI m/z (%): 65 (11.7), 91 (100), 115 (12.6), 142 (80.1), 143 (12.9), 241 (11.8), 242 (30.3), 332 (16.1), 333 (41.4), 368 (80.2), 370 (31.9); HRMS (EI) calcd for C_19_H_17_N_4_SCl (M): 368.0862 (80.2 %). Found: 368.0857, M + 2: 370.0 (31.9).

#### 3-Benzylsulfanyl-2-(3-bromopropyl)-5-(1*H*-indol-2-yl) -1,2,4-triazole (8)

Colorless crystals, Yield 60 %; m.p. 96–97 °C; R_*f*_ 0.66 (ethyl acetate/n-hexane 4:6); ^1^H NMR (DMSO-*d*_6_, 600 MHz) δ 2.48–2.50 (m, 2H, CH_2_), 3.74 (t, 2H, *J* = 5.4 Hz, CH_2_), 4.42 (t, 2H, *J* = 6.6 Hz, CH_2_), 4.81 (s, 2H, C*H*_2_Ph), 7.22 (s, 1H, H-3_Indol_), 7.30 (dd, 1H, *J*_4,5_ = 7.8, *J*_5,6_ = 7.2 Hz, H-5_Indol_), 7.42 (dd, 1H, *J*_5,6_ = 7.2, *J*_6,7_ = 8.4 Hz, H-5_Indol_), 7.54–7.87 (m, 7H, H-4_Indol_, H-7_Indol_, 5H_Ph_), 11.91 (s, 1H, NH_Indol_); ^13^C NMR (DMSO-*d*_6_, 150 MHz) δ 31.0 (CH_2_), 31.9 (CH_2_), 37.4 (*C*H_2_Ph), 46.6 (NCH_2_), 100.6 (C-3_Indol_), 111.8 (C-7_Indol_), 119.5 (C-5_Indol_), 120.5 (C-4_Indol_), 122.2 (C-6_Indol_), 127.6, 127.8, 128.5, 128.6, 128.7, 128.9, 129.1, 128.2, 136.8, 137.2 (C-2_Indol_, C-3a_Indol_, 5CH_Ph_, C-7a_Indol_, C_Ph_), 151.4 (C-5_Triazol_), 156.2 (C-3_Triazol_); HRMS (ESI) calcd for C_20_H_20_BrN_4_S (M + H)^+^: 427.0592 Found: 427.0585 (M + H)^+^, 429 (100 %) for (M + H+2)^+^.

#### 1*H*-Indolo[1,2-a]-3-phenylsulfanyl-1,2,4-triazolo[1,5-c]1,4-diazepine (10)

Colorless crystals, Yield 28 %; m.p. 148–149 °C; R_*f*_ 0.35 (ethyl acetate/n-hexane 4:6); ^1^H NMR (DMSO-*d*_6_, 600 MHz) δ 2.43–2.48 (m, 2H, CH_2_), 4.39 (s, 2H, CH_2_Ph), 4.45–4.54 (m, 4H, 2 CH_2_), 7.12 (dd, 1H, *J*_4,5_ = 7.8, *J*_5,6_ = 7.2 Hz, H-5_Indol_), 7.24–7.32 (m, 5H, H-3_Indol_, H-6_Indol_, 3H_Ph_), 7.44 (d, 2H, *J* = 7.2 Hz, 2H_Ph_), 7.56 (d, 1H, *J*_6,7_ = 8.4 Hz, H-7_Indol_), 7.64 (d, 1H, *J*_4,5_ = 7.8 Hz, H-4_Indol_); ^13^C NMR (DMSO-*d*_6_, 150 MHz) δ 25.2 CH_2_), 35.0 (*C*H_2_Ph), 44.7 (CH_2_), 51.7 (CH_2_), 105.7 (C-3_Indol_), 110.4 (C-7_Indol_), 120.3 (C-5_Indol_), 121.2 (C-4_Indol_), 123.3 (C-6_Indol_), 126.5, 126.7, 127.2 (C-2_Indol_, C-3a_Indol_, CH_Ph_), 128.4 (2 CH_Ph_), 128.9 (2 CH_Ph_), 137.9, 137.6 (C-7a_Indol_, C_Ph_), 148.6 (C-5_Triazol_), 158.5 (C-3_Triazol_); HRMS (EI) calcd for C_20_H_18_N_4_S (M^+.^): 346.1252. Found: 346.1243.

#### 2,3-Bis((3-benzylsulfanyl-5-(1*H*-indol-2-yl)-1,2,4-triazol-1-yl)methyl)quinoxaline (11)

White solid, Yield 20 %; m.p. 175–176 °C; R_*f*_ 0.41 (ethyl acetate/n-hexane 3:7); ^1^H NMR (CDCl_3_, 300 MHz) δ 4.34 (s, 4 H, 2 SCH_2_Ph), 6.14 (s, 4 H, 2 NCH_2 quinoxalin_), 7.11–7.39 (m, 16 H, 2 H-3_Indol_, 2 H-5_Indol_, 2 H-6_Indol_, 10 H, 2Ph), 7.57 (d, 2 H, *J*_6,7_ 9.0 Hz, 2 H-4_Indol_), 7.76 (d, 2 H, *J*_4,5_ 9.0 Hz, 2 H-7_Indol_), 7.87–7.91 (m, 2 H, 2 CH_quinoxalin_), 8.15–8.18 (m, 2 H, 2 CH_quinoxalin_), 10.56 (s, 2 H, NH_Indol_); ^13^C NMR (CDCl_3_, 100 MHz) δ 36.44 (2 CH_2_ph), 51.97 (2 CH_2 quinoxalin_), 105.98 (2 C-3_Indol_), 111.80 (2 C-7_Indol_), 120.96 (2 C-5_Indol_), 122.04 (2 C-4_Indol_), 122.04(2 C-2_Indol_), 124.60 (2 C-6_Indol_), 127.41, 128.38, 128.52, 128.97, 129.01, 131.77, 137.13, 137.24 (2 C-3a_Indol_, 10 CH_ph_, 2C_Ph_, 4 CH_quinoxalin_, 2 C-7a_Indol_), 141.41, 149.05, 150.16, 160.33 (2 C_quinoxalin_, 2 C-3_Triazol_, 2 C-5_Triazol_); ESI^+^-MS m/z (rel. abundance  %): 119.7 (8), 148.9 (17), 301.0 (14), 360.2 (9), 413.1 (8), 663.4 (6), 767.3 (28) (M + H)^+^ for C_44_H_35_N_10_S_2_, 789.2 (45) (M + Na)^+^, 805.2 (100) (M + K)^+^.

#### 2,3-Bis((3-benzylsulfanyl-5-(1*H*-indol-2-yl)-1,2,4-triazol-2-yl)methyl)quinoxaline (12)

White solid, Yield 50 %; m.p. 117–118 °C; R_*f*_ 0.65 (ethyl acetate/n-hexane 3:7); ^1^H NMR (DMSO-*d*_6_, 600 MHz) δ 5.22 (s, 4 H, 2 SCH_2_-Ph), 5.42 (s, 4 H, 2 NCH_2__quinoxalin_), 6.90 (s, 2 H, 2 H-3_Indol_), 6.97 (dd, 2 H, *J*_4,5_ 7.8, *J*_5,6_ 7.2, Hz, 2 H-5_Indol_), 7.10 (dd, 2 H, *J*_5,6_ 7.2, *J*_6,7_ 7.8 Hz, 2 H-6_Indol_), 7.21–7.26 (m, 10 H, 2Ph), 7.39 (d, 2 H, *J*_6,7_ 8.1 Hz, 2 H-4_Indol_), 7.46 (d, 2 H, *J*_4,5_ 7.5 Hz, 2 H-7_Indol_), 7.82–7.34 (m, 2 H, 2 CH_quinoxalin_), 7.99–8.01 (m, 2 H, 2 CH_quinoxalin_), 11.60 (s, 2 H, NH_Indol_); ^13^C NMR (DMSO-*d*_6_, 150 MHz) δ 37.9 (2 CH_2_ph), 52.3 (2 CH_2 quinoxalin_), 101.5 (2C-3_Indol_), 112.3 (2C-7_Indol_), 119.9 (2C-5_Indol_), 121.0 (2C-4_Indol_), 122.72 (2C-6_Indol_), 127.66, 127.72, 127.74, 128.2, 128.4, 128.75, 129.1, 129.4, 129.5, 130.9, 131.9, 136.1, 137.4 (2 C-2_Indol_, 2 C-3a_Indol_, 10 CH_ph_, 2C_Ph_, 4 CH_quinoxalin_, 2 C-7a_Indol_), 140.7, 151.4, 152.4, 156.8 (2 C_quinoxalin_, 2 C-3_Triazol_, 2 C-5_Triazol_); ESI^+^-MS m/z (rel. abundance  %): 148.9 (18), 239.1 (7), 276.0 (27), 301.0 (20), 344.0 (12), 413.0 (11), 540.1 (6), 633.5 (8), 767.2 (35) (M + H)^+^ for C_44_H_35_N_10_S_2_, 789.2 (87) (M + Na)^+^, 805.2 (100) (M + K)^+^.

## Additional file

10.1186/s13065-016-0165-0 Chemical information files (cif) of compounds **4**, **6**, **8** and **10**.
10.1186/s13065-016-0165-0 Copies of the NMR spectra of new synthesized compounds.
