# Supplementary material for: Regioselectivity of the alkylation of S-substituted 1,2,4-triazoles with dihaloalkanes
Source: Chem Cent J. 2016 Apr 27;10:22. doi: 10.1186/s13065-016-0165-0 (PMC4849090; doi:10.1186/s13065-016-0165-0)
Supplement: Supplementary file 2 — 10.1186/s13065-016-0165-0 Copies of the NMR spectra of new synthesized compounds. [file 13065_2016_165_MOESM2_ESM.pdf]

Electronic Supplementary Information to

Regioselectivity of the alkylation of S-substituted 1,2,4-triazoles with dihaloalkanes

Ahmed T.A. Boraei,[a*] El Sayed H. El Ashry,[b*] Axel Duerkop[c]

[a]*Chemistry Department, Faculty of Science, Suez Canal University, Ismailia 41522, Egypt*

[b]*Chemistry Department, Faculty of science, Alexandria University, Alexandria, Egypt*

[c]*Institute of Analytical Chemistry, Chemo and Biosensors, Universitätsstrasse 31, 93053 Regensburg, Germany*

**Figure number and description**	**Page number**
Figure 1. ^{1}H NMR of Compound **2**	**3**
Figure 2. ^{13}C NMR of Compound **2**	**4**
Figure 3. ^{1}H NMR of compound **3**	**5**
Figure 4. ^{13}C NMR of Compounds **3**	**6**
Figure 5. ^{1}H NMR of compound **4**	**7**
Figure 6. ^{13}C NMR of compound **4**	**8**
Figure 7. HMBC of compound **4**	**9**
Figure 8. ^{1}H NMR of compound **5**	**10**
Figure 9. ^{13}C NMR of compound **5**	**11**
Figure 10. HMBC of compound **5**	**12**
Figure 11. ^{1}H NMR of compound **6**	**13**
Figure 12. ^{13}C NMR of compound **6**	**14**
Figure 13. HMBC of compound **6**	**15**
Figure 14. ^{1}H NMR of compound **7**	**16**
Figure 15. ^{13}C NMR of compound **7**	**17**
Figure 16. ^{1}H NMR of compound **8**	**18**
Figure 17. ^{13}C NMR of compound **8**	**19**
Figure 18. ^{1}H NMR of compound **10**	**20**
Figure 19. ^{13}C NMR of compound **10**	**21**
Figure 20. ^{1}H NMR of compound **11**	**22**
Figure 21. ^{13}C NMR of compound **11**	**23**
Figure 22. ^{1}H NMR of compound **12**	**25**
Figure 23. ^{13}C NMR of compound **12**	**25**

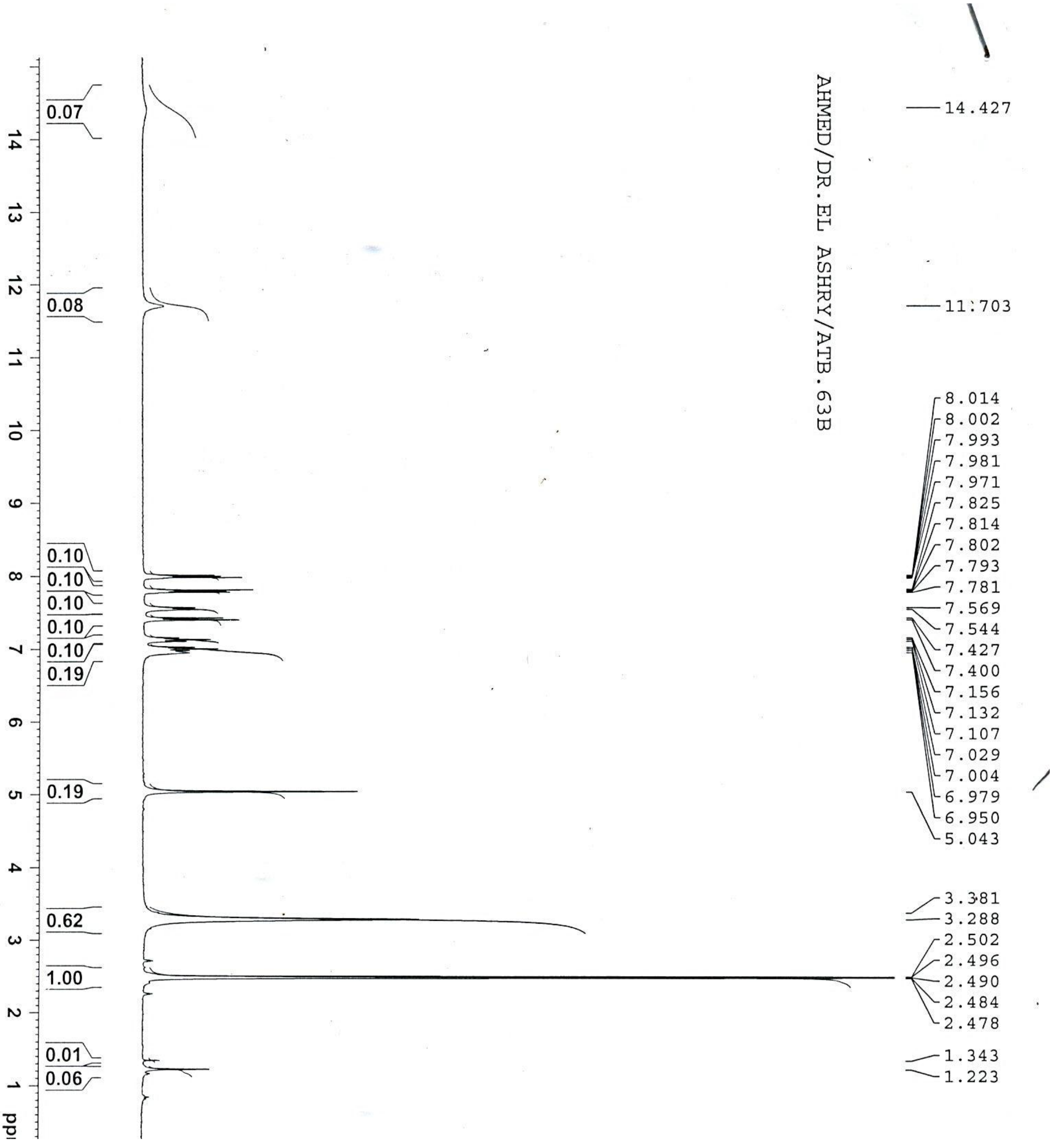

Figure 1. ^{1}H NMR of Compound **2**

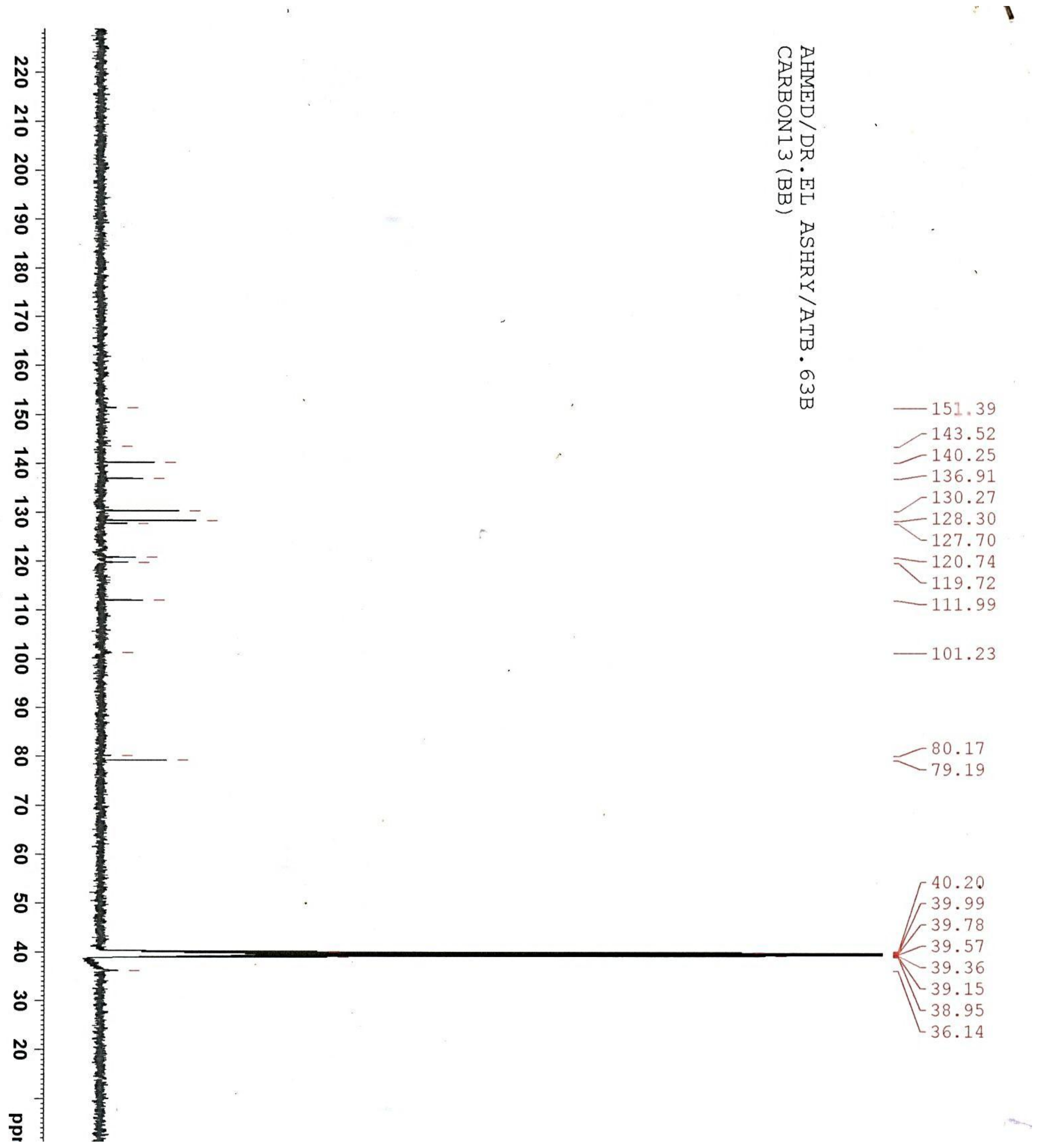

Figure 2. ^{13}C NMR of Compound **2**

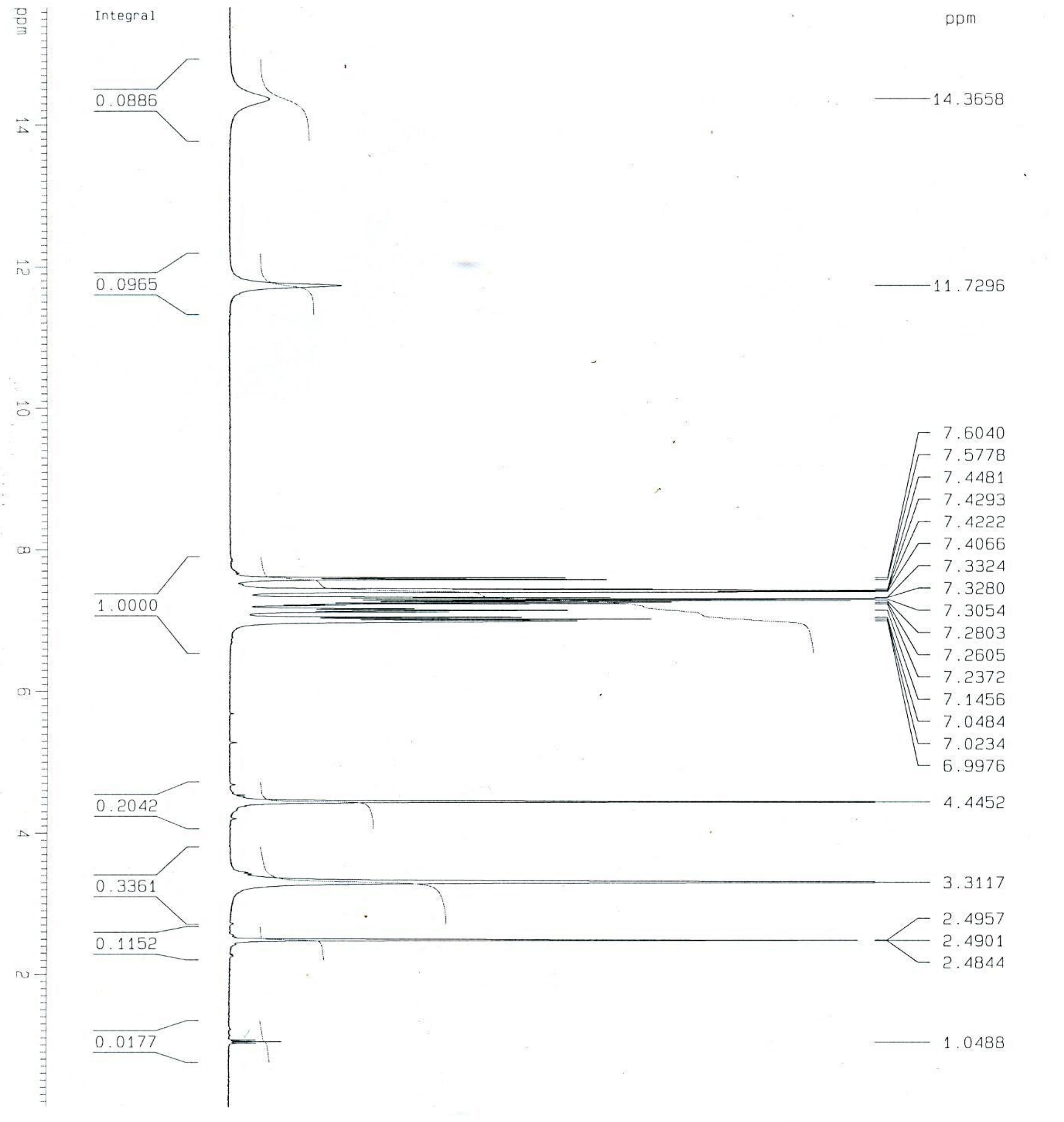

Figure 3. ^{1}H NMR of compound **3**

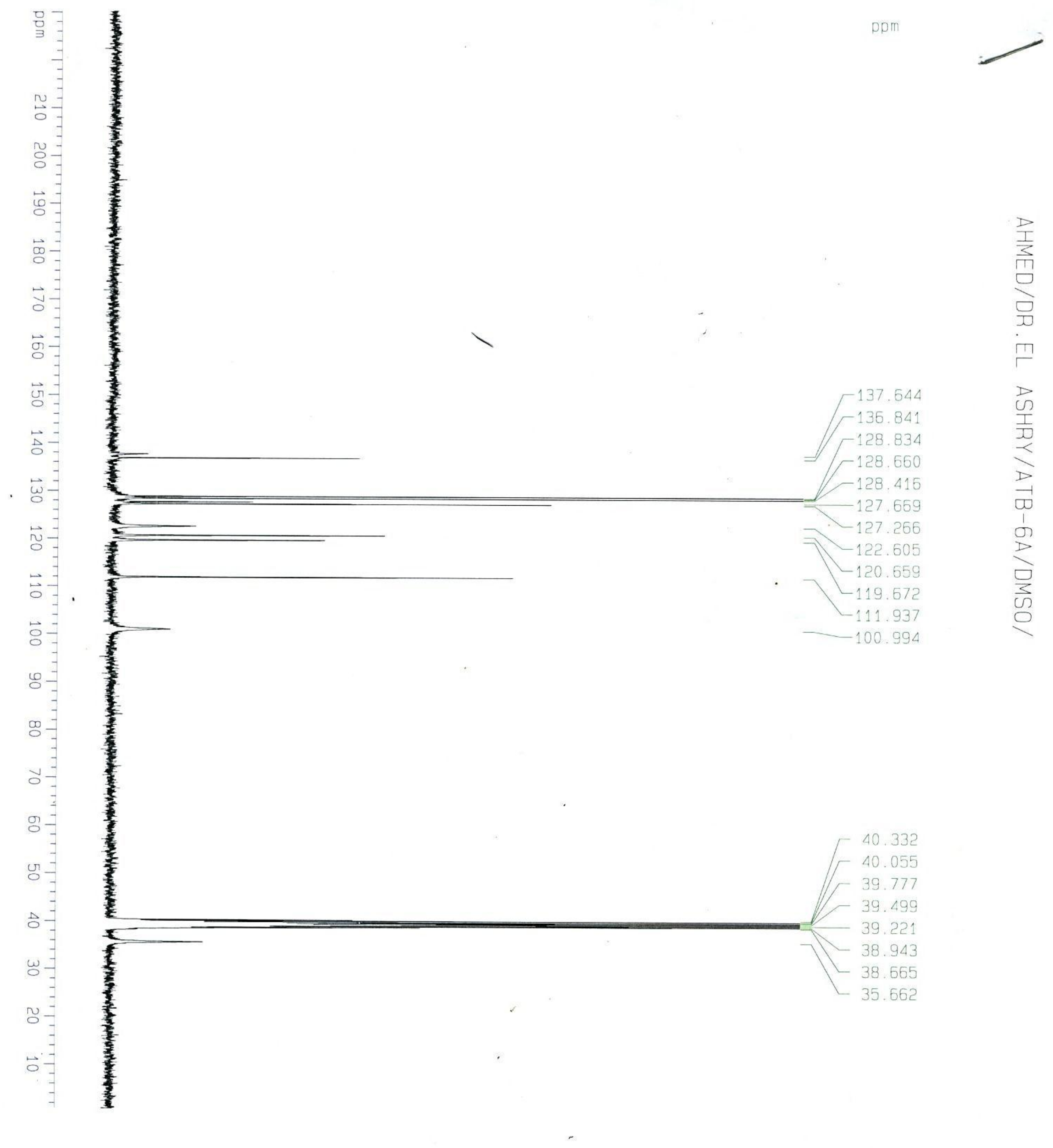

Figure 4. ^{13}C NMR of Compounds **3**

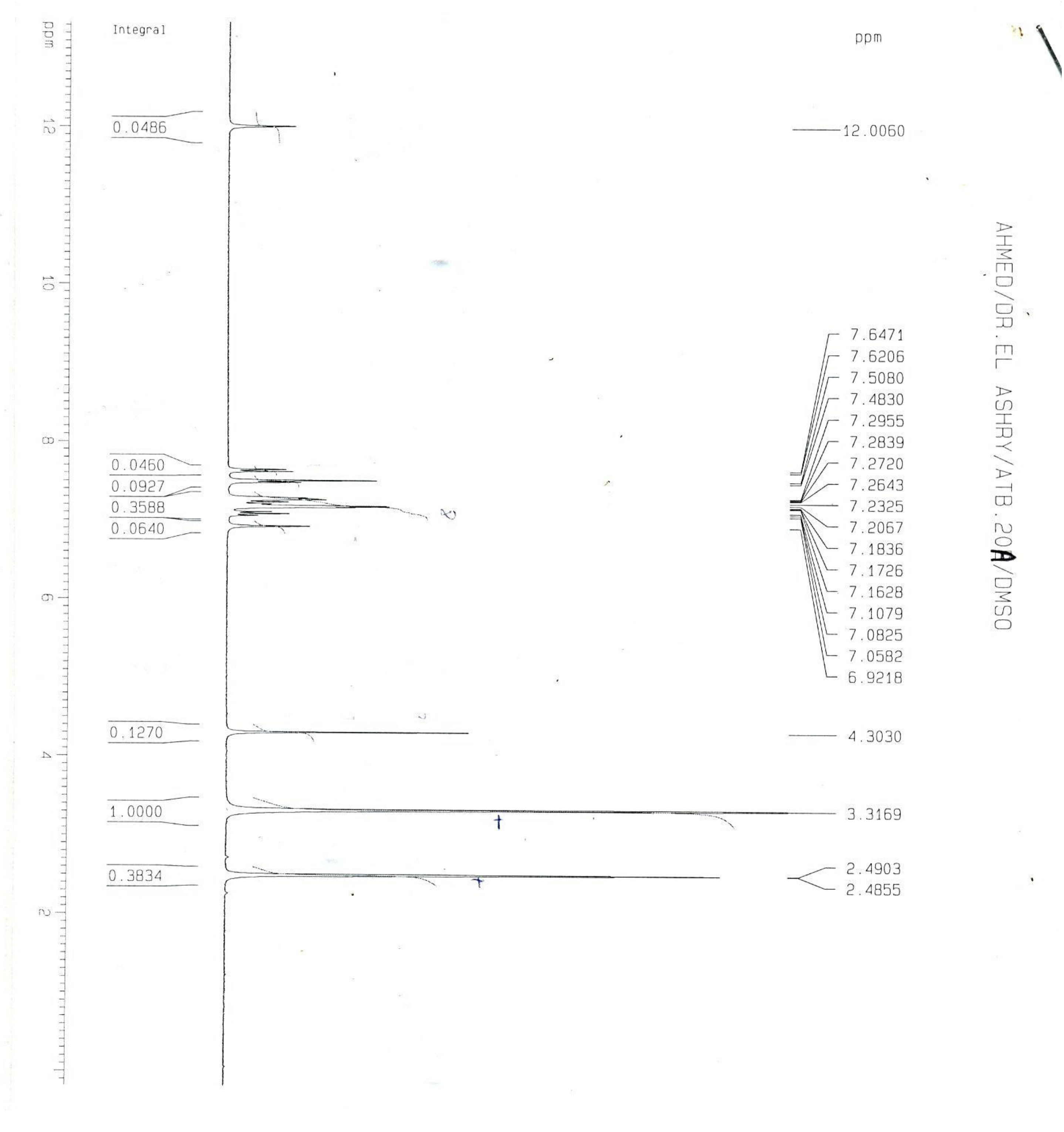

Figure 5. ^{1}H NMR of compound **4**

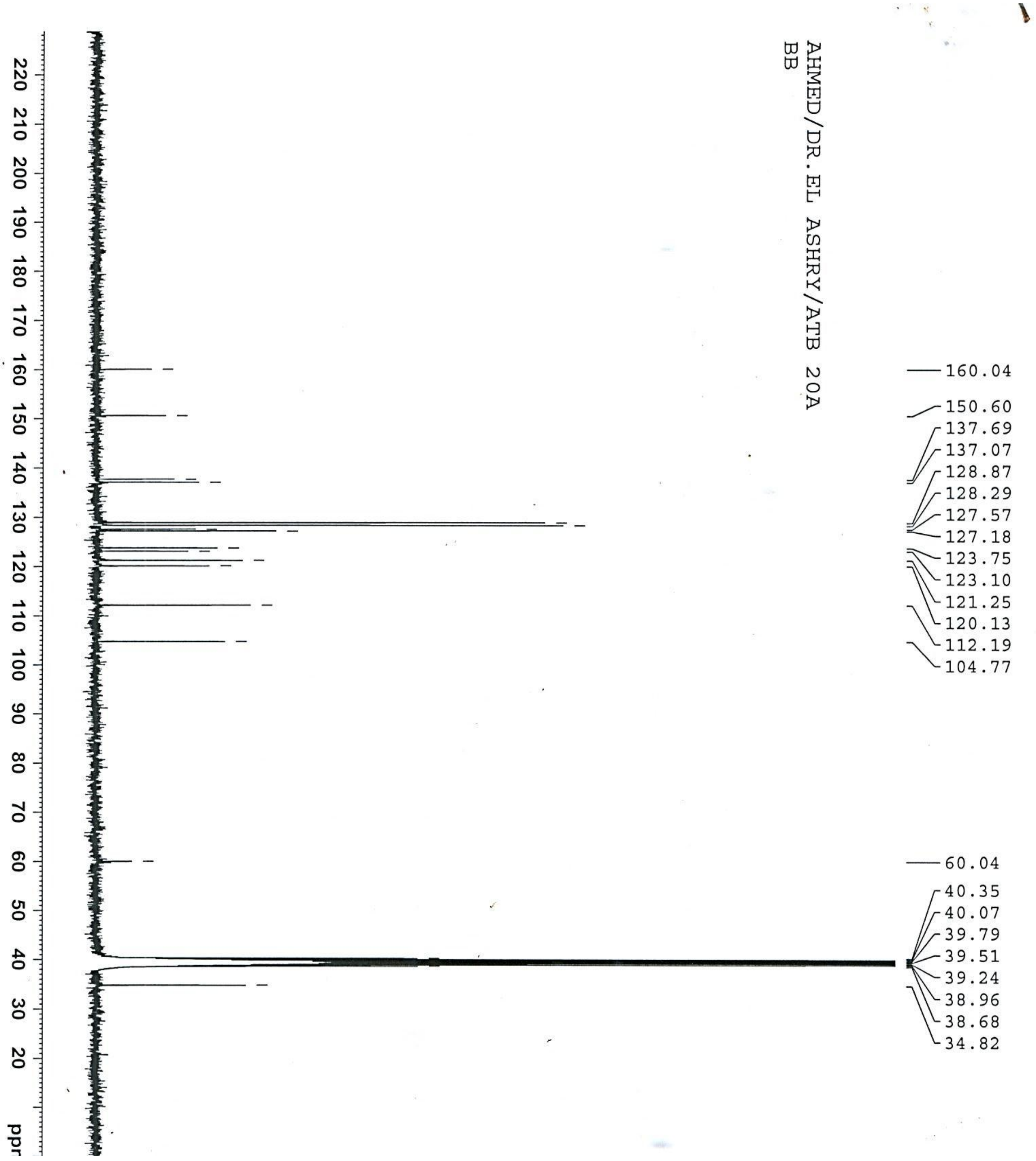

Figure 6. ^{13}C NMR of compound **4**

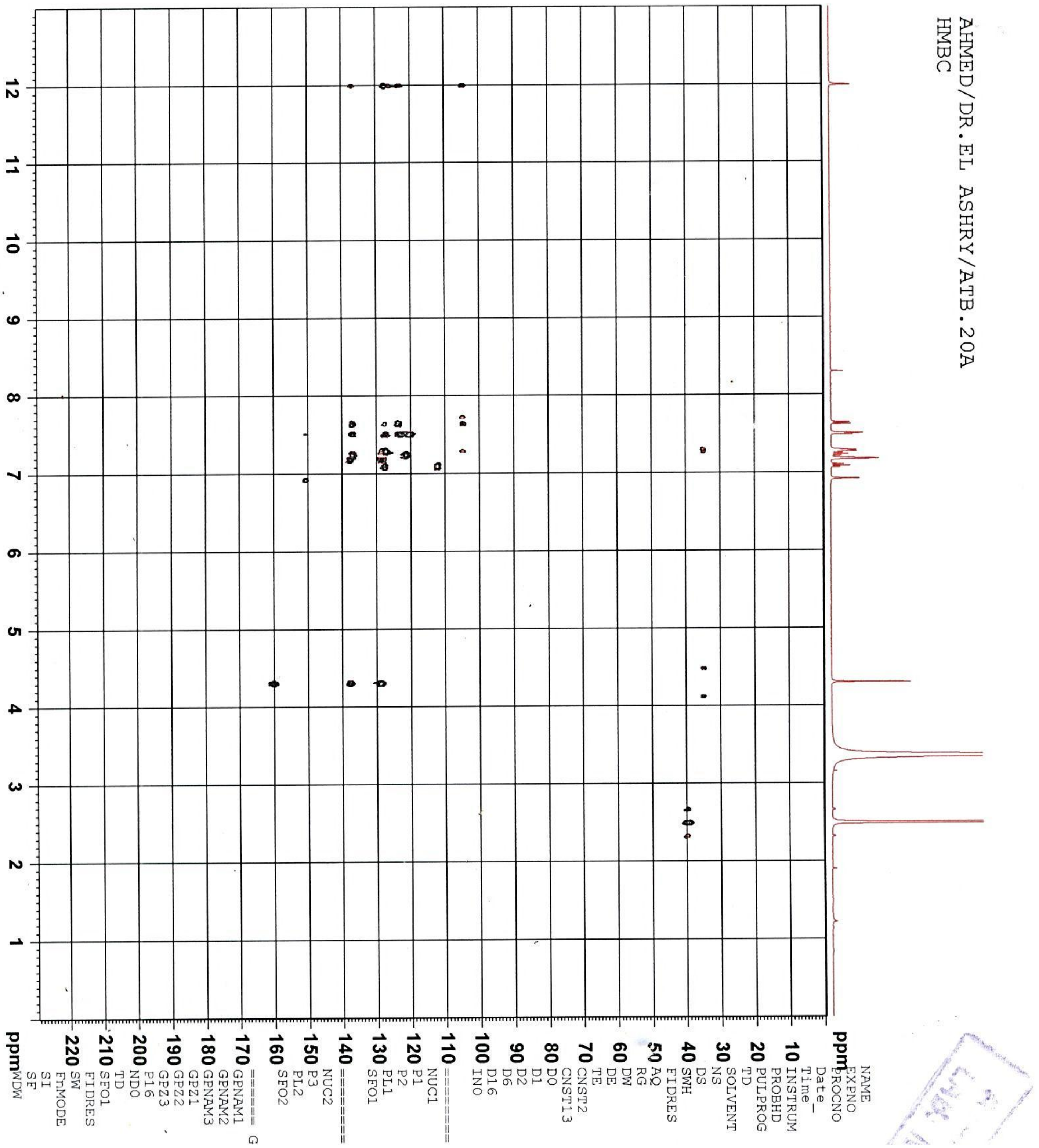

Figure 7. HMBC of compound **4**

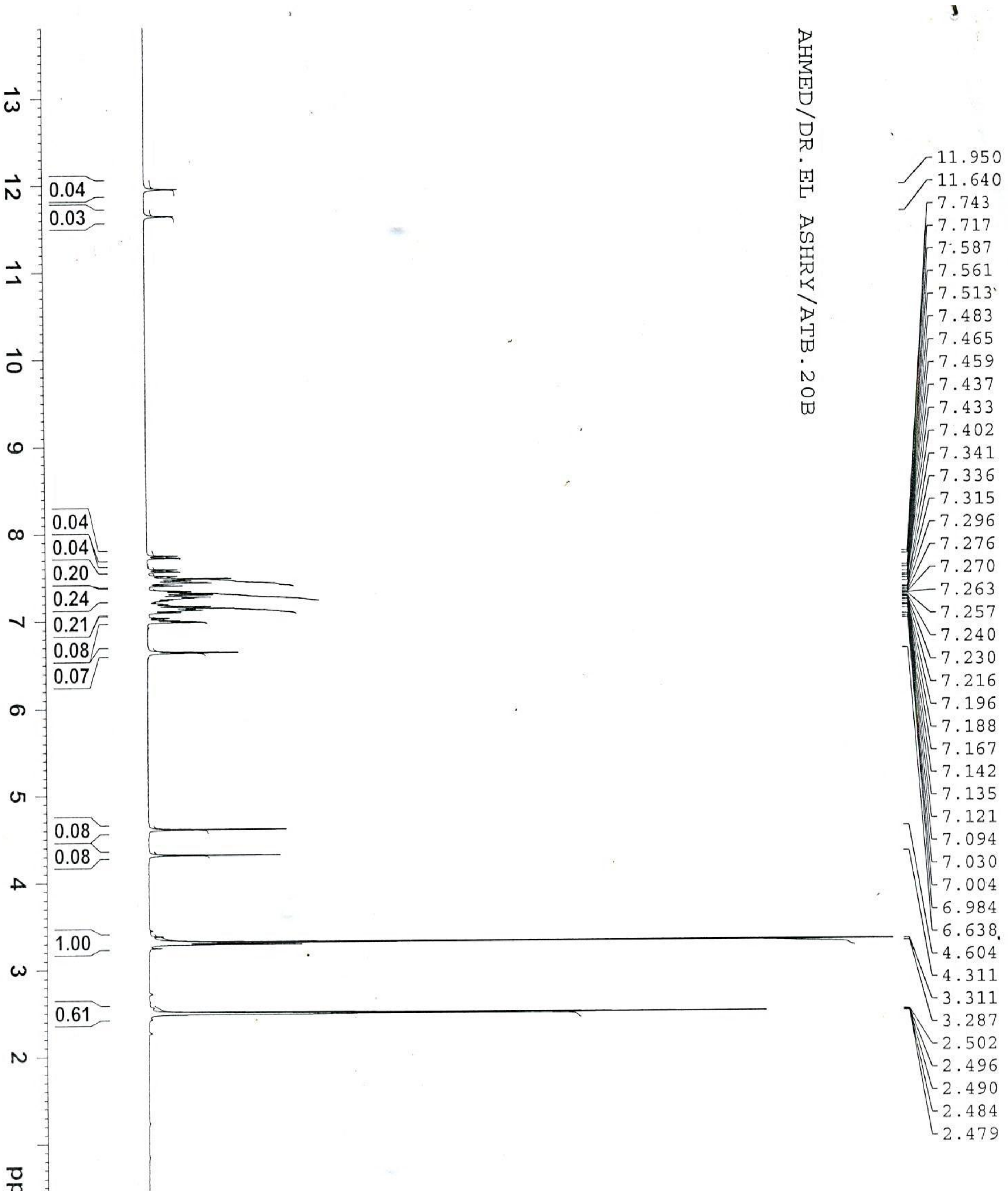

Figure 8. ^{1}H NMR of compound **5**

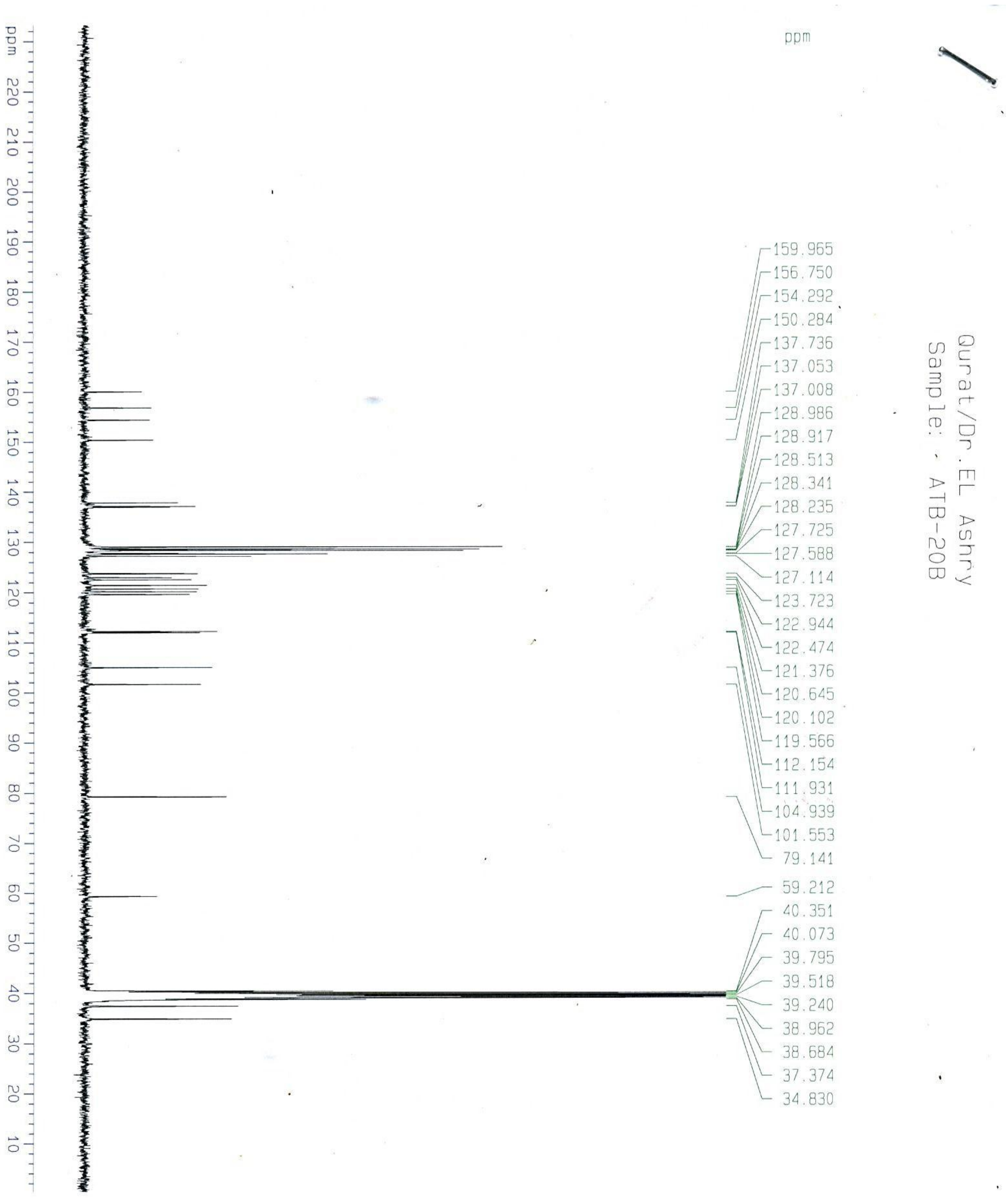

Figure 9. ^{13}C NMR of compound **5**

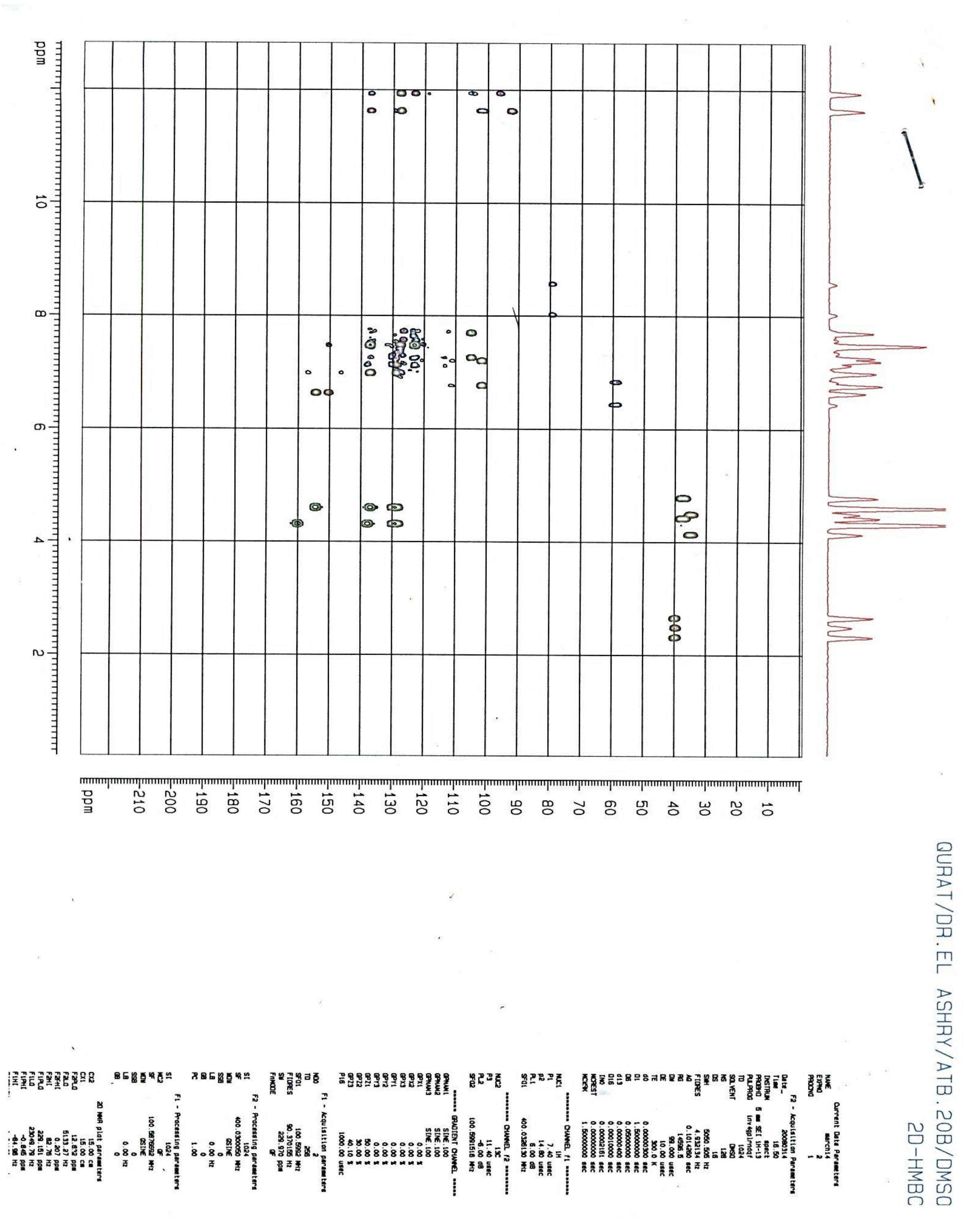

Figure 10. HMBC of compound **5**

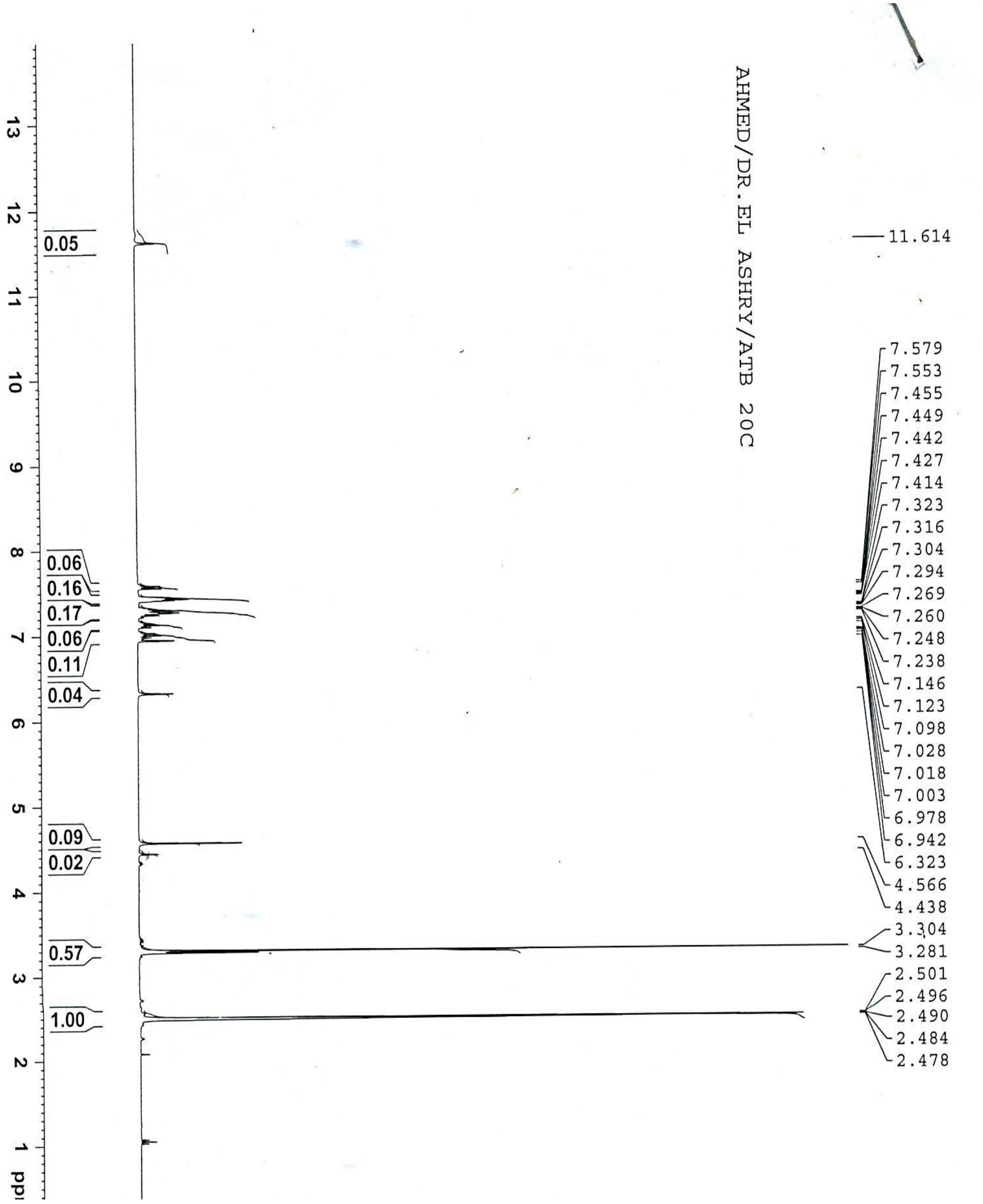

Figure 11. ^{1}H NMR of compound **6**

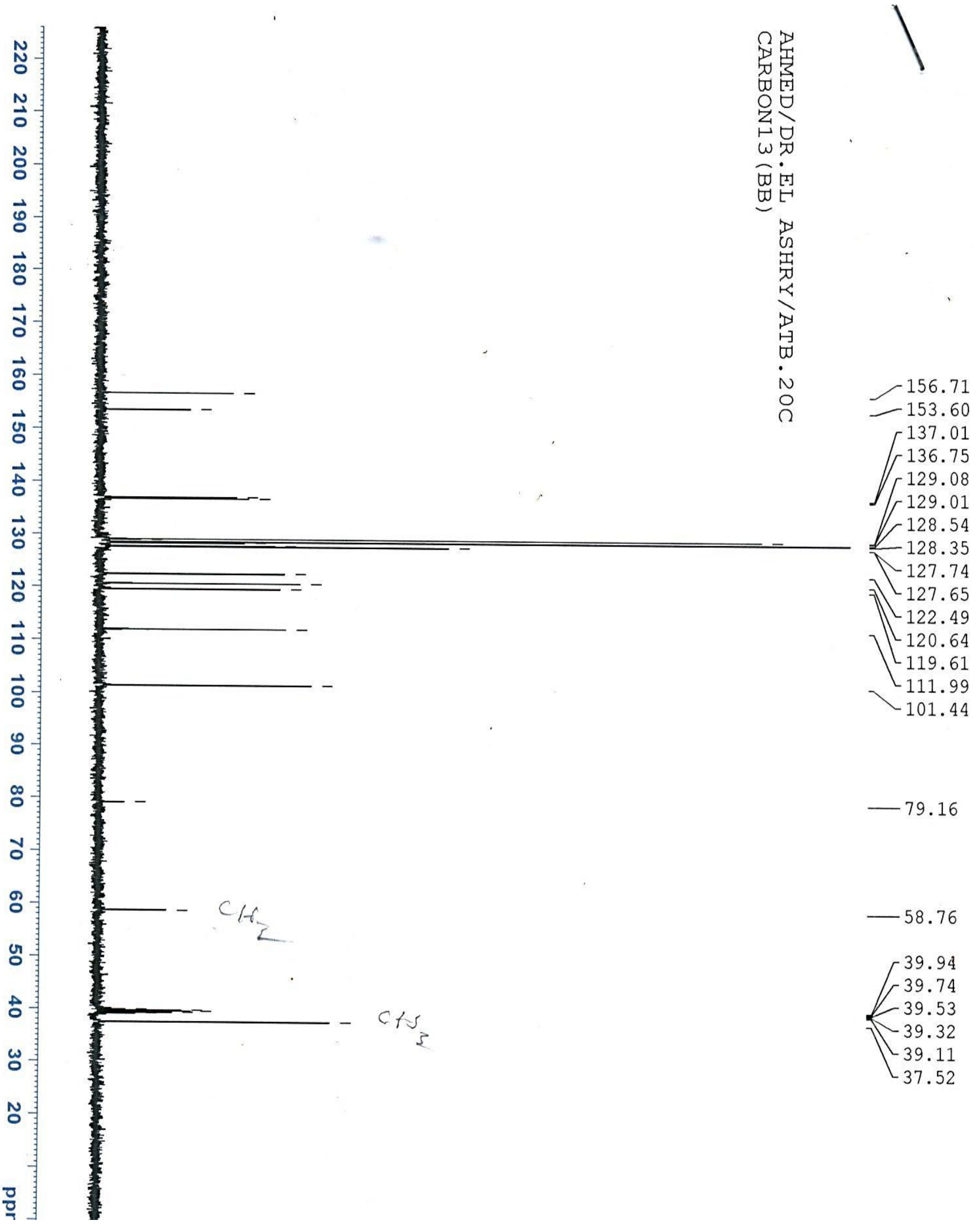

Figure 12. ^{13}C NMR of compound **6**

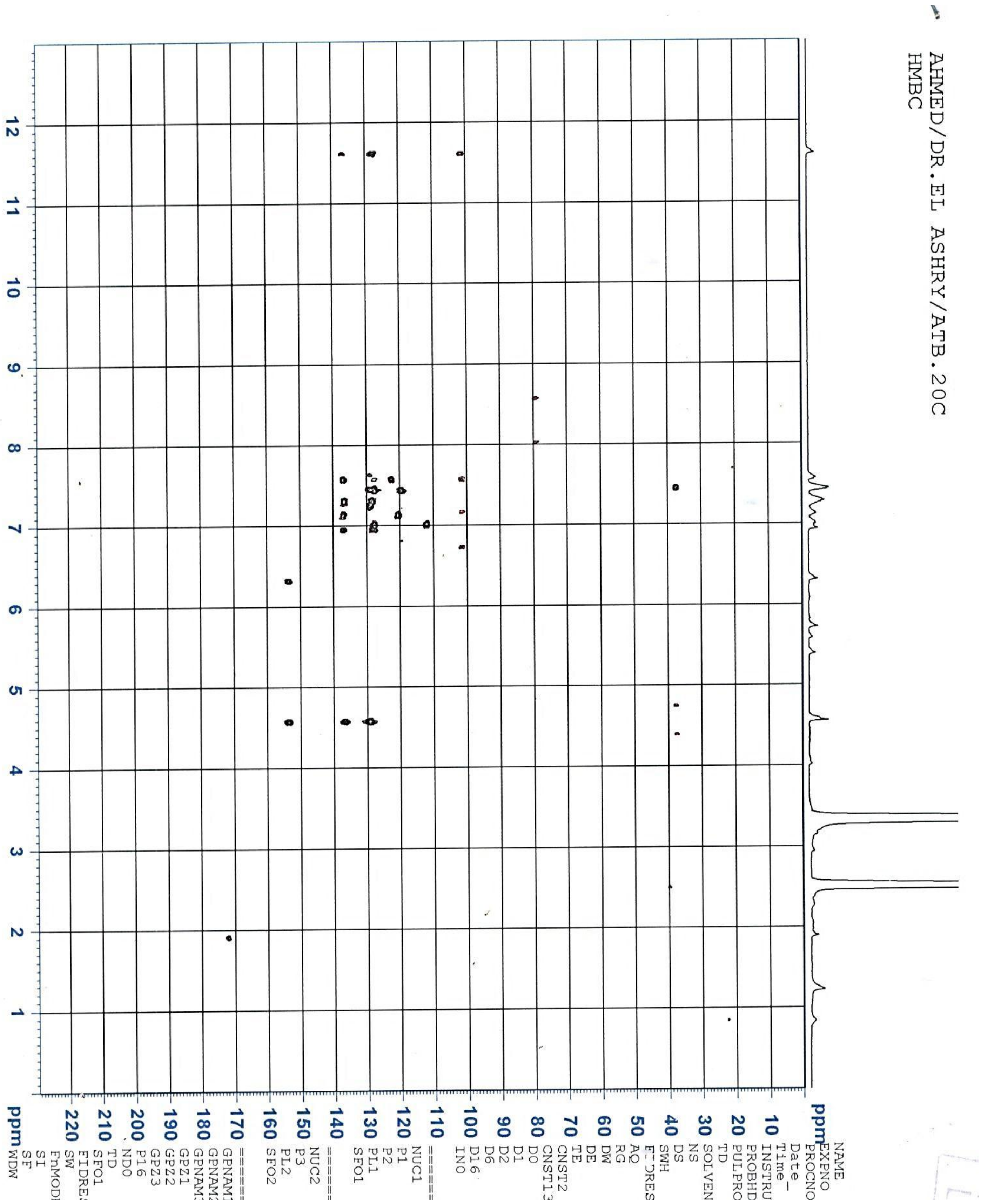

Figure 13. HMBC of compound **6**

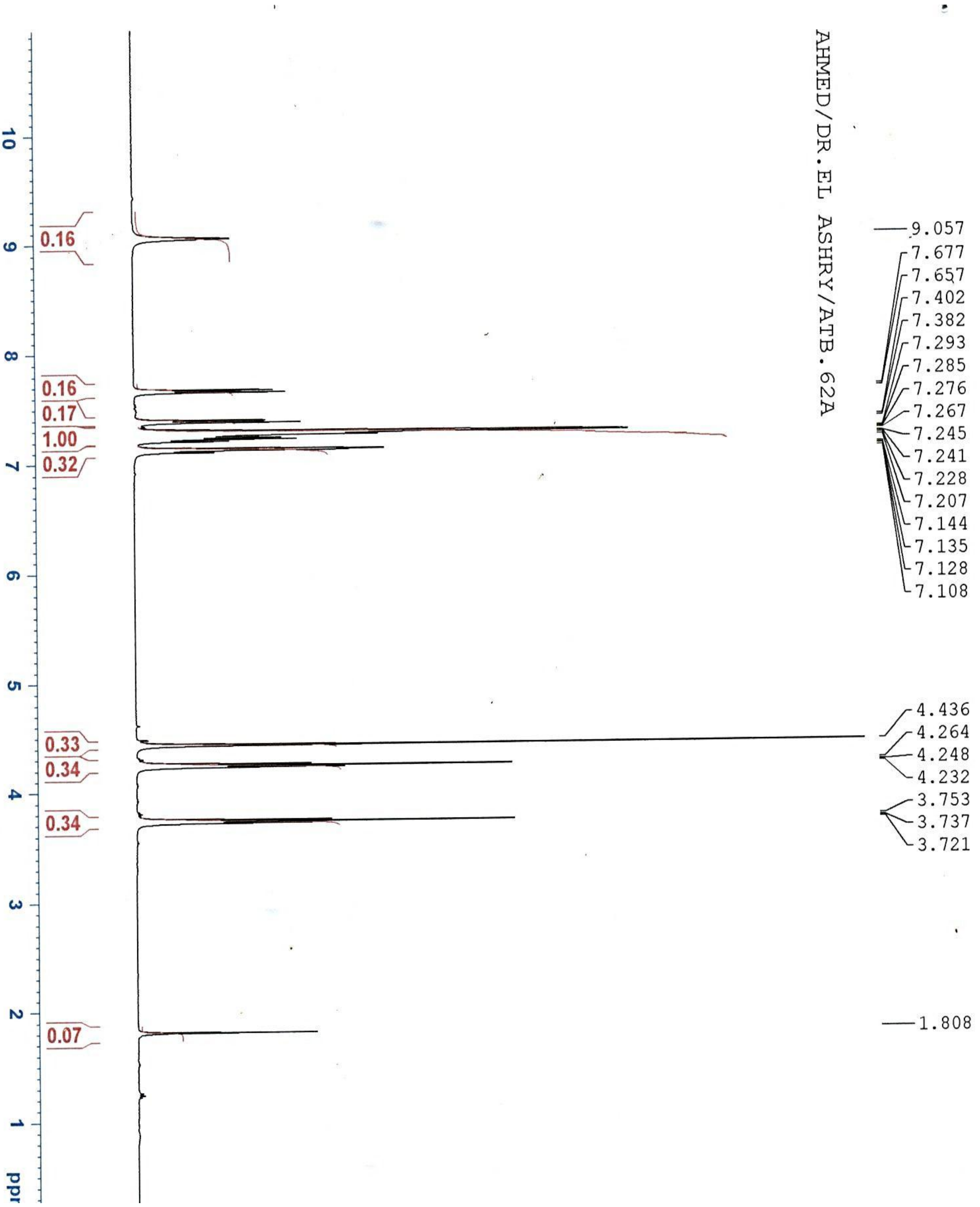

Figure 14. ^{1}H NMR of compound **7**

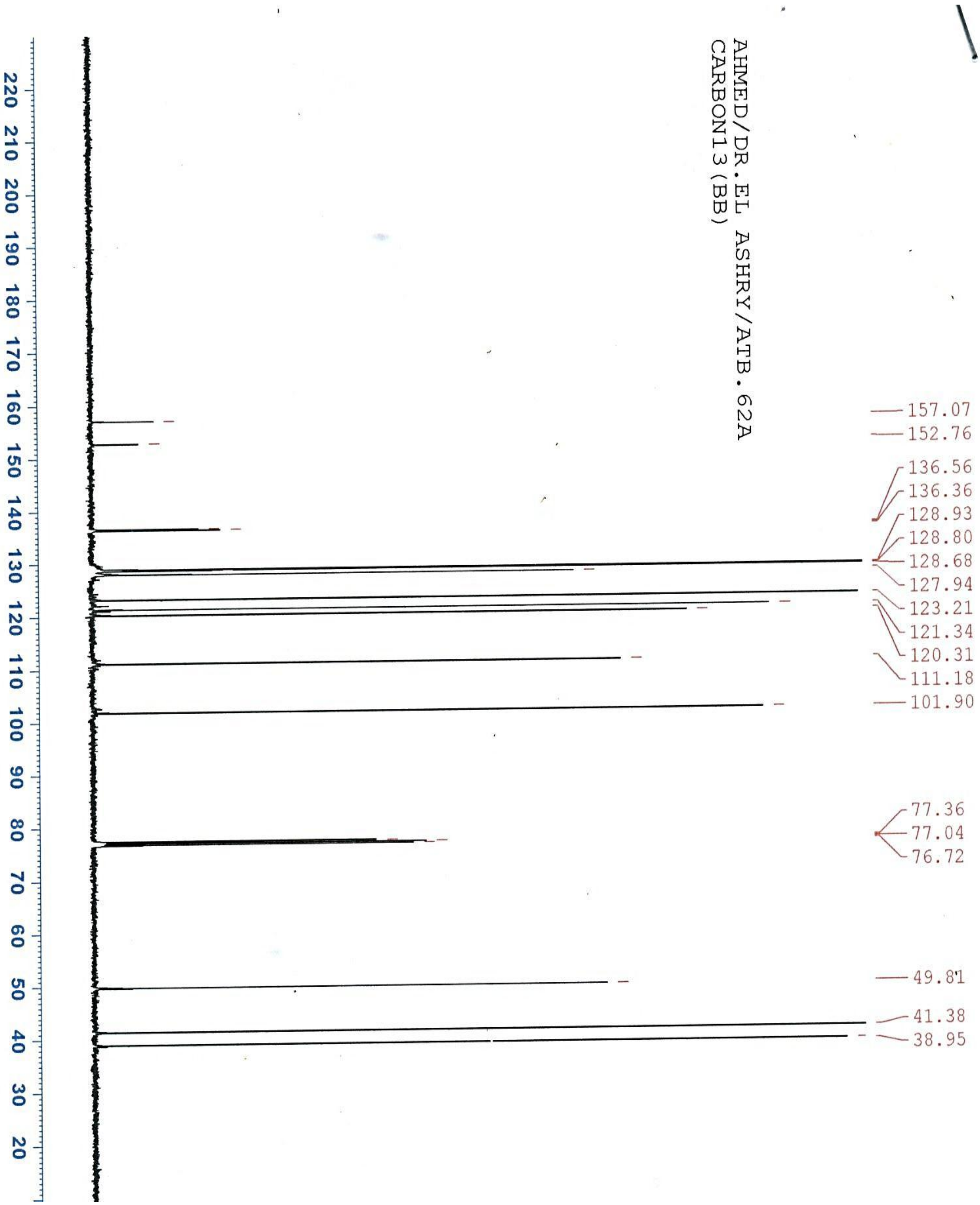

Figure 15. ^{13}C NMR of compound **7**

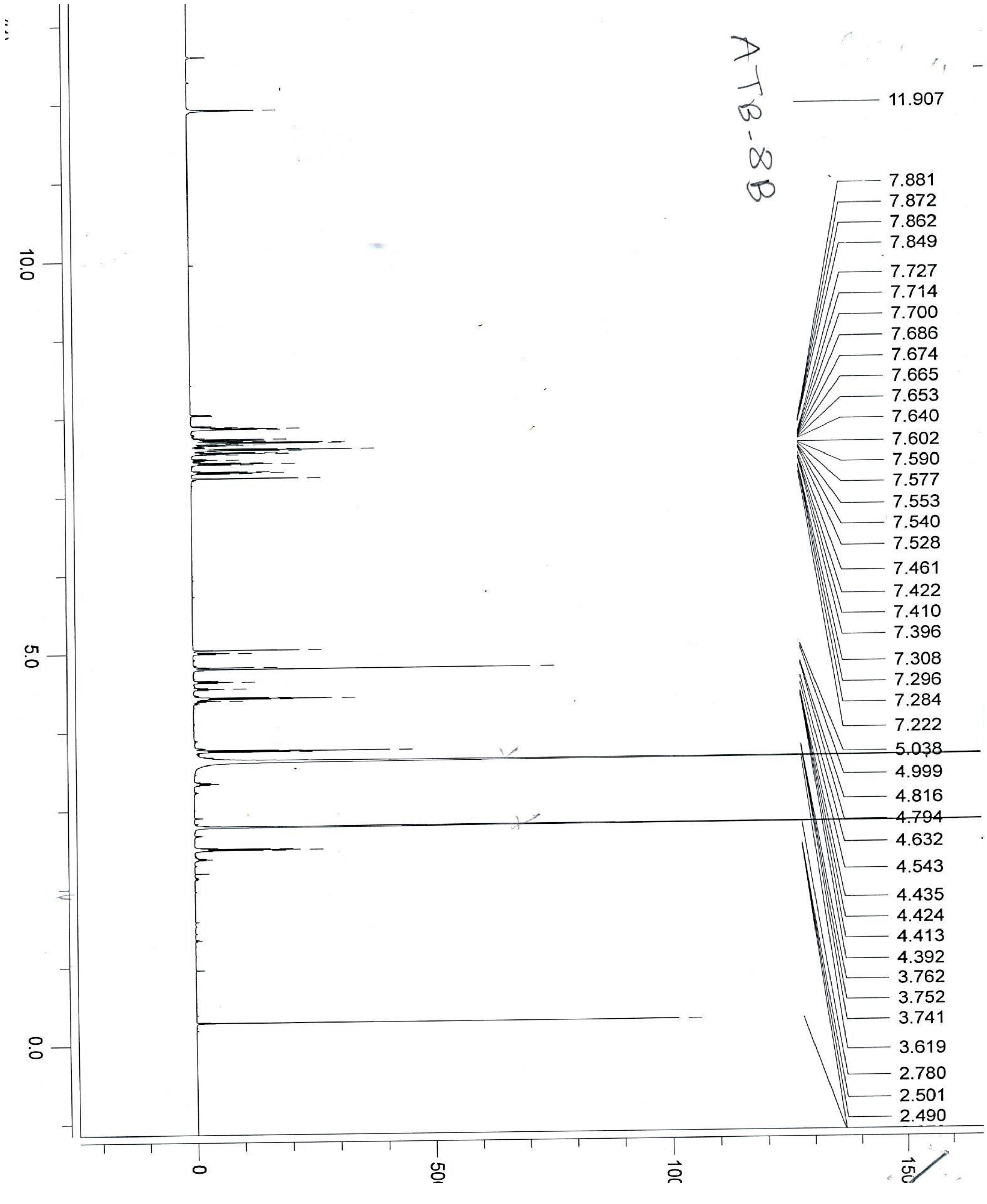

Figure 16. ^{1}H NMR of compound **8**

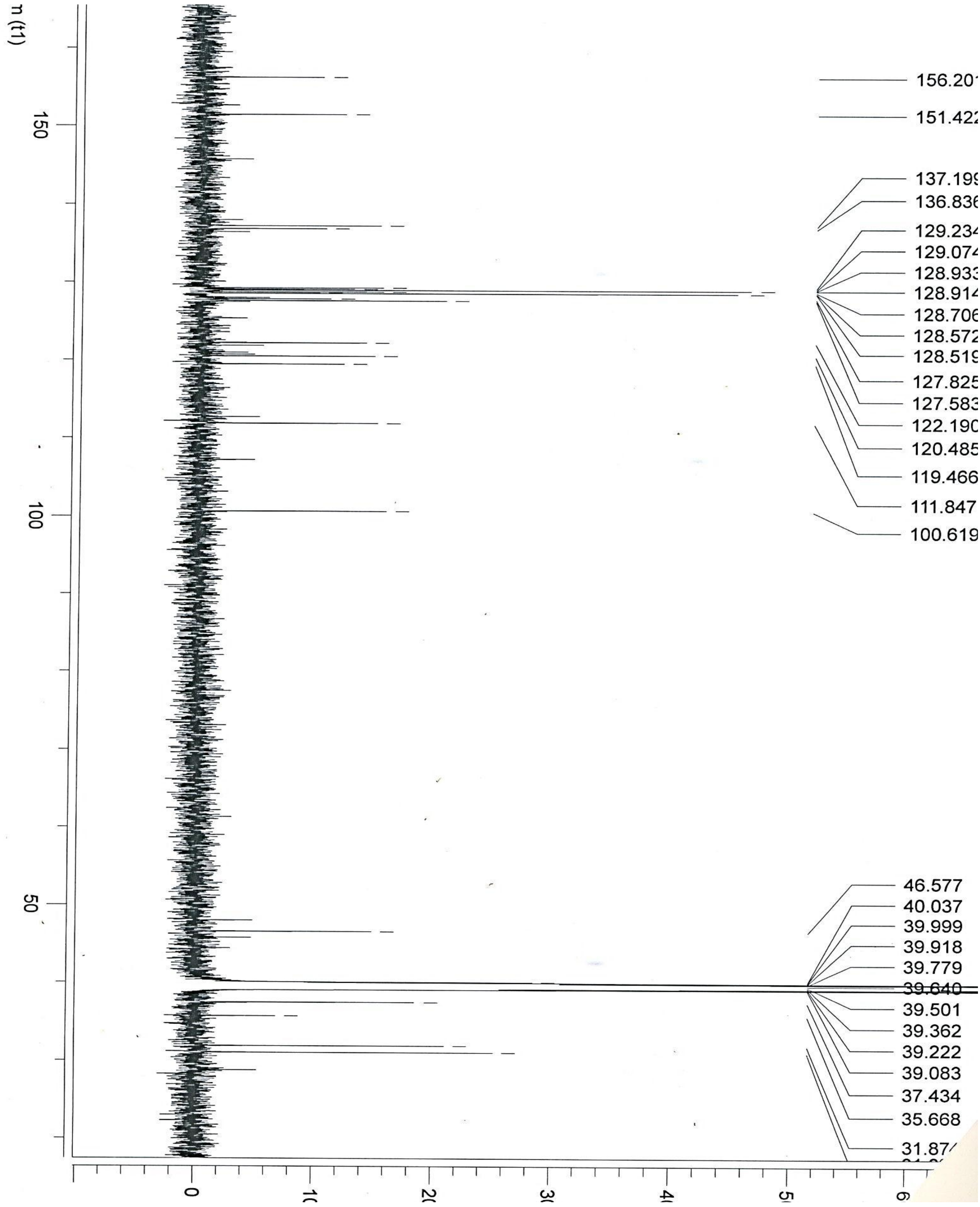

Figure 17. ^{13}C NMR of compound **8**

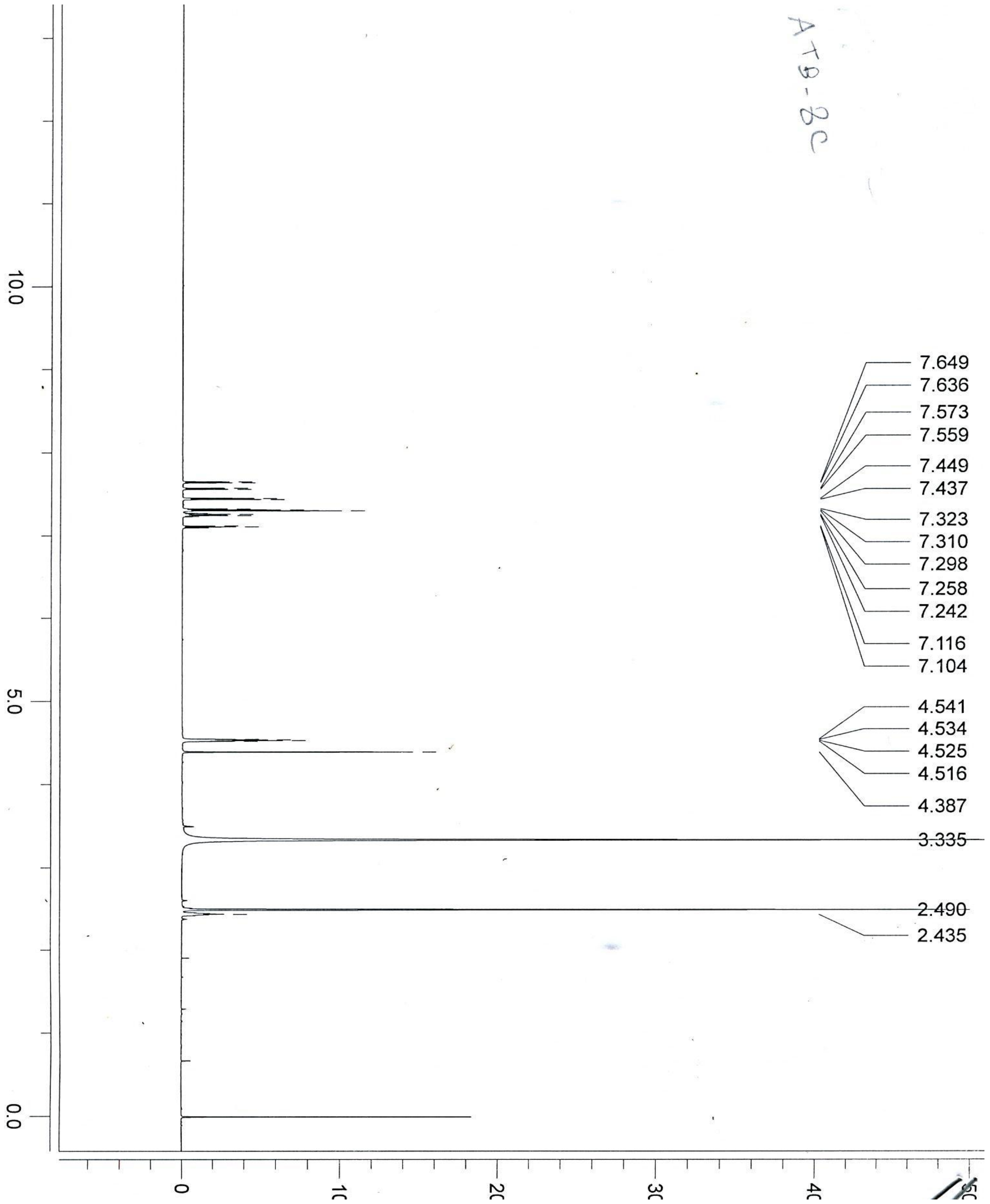

Figure 18. ^{1}H NMR of compound **10**

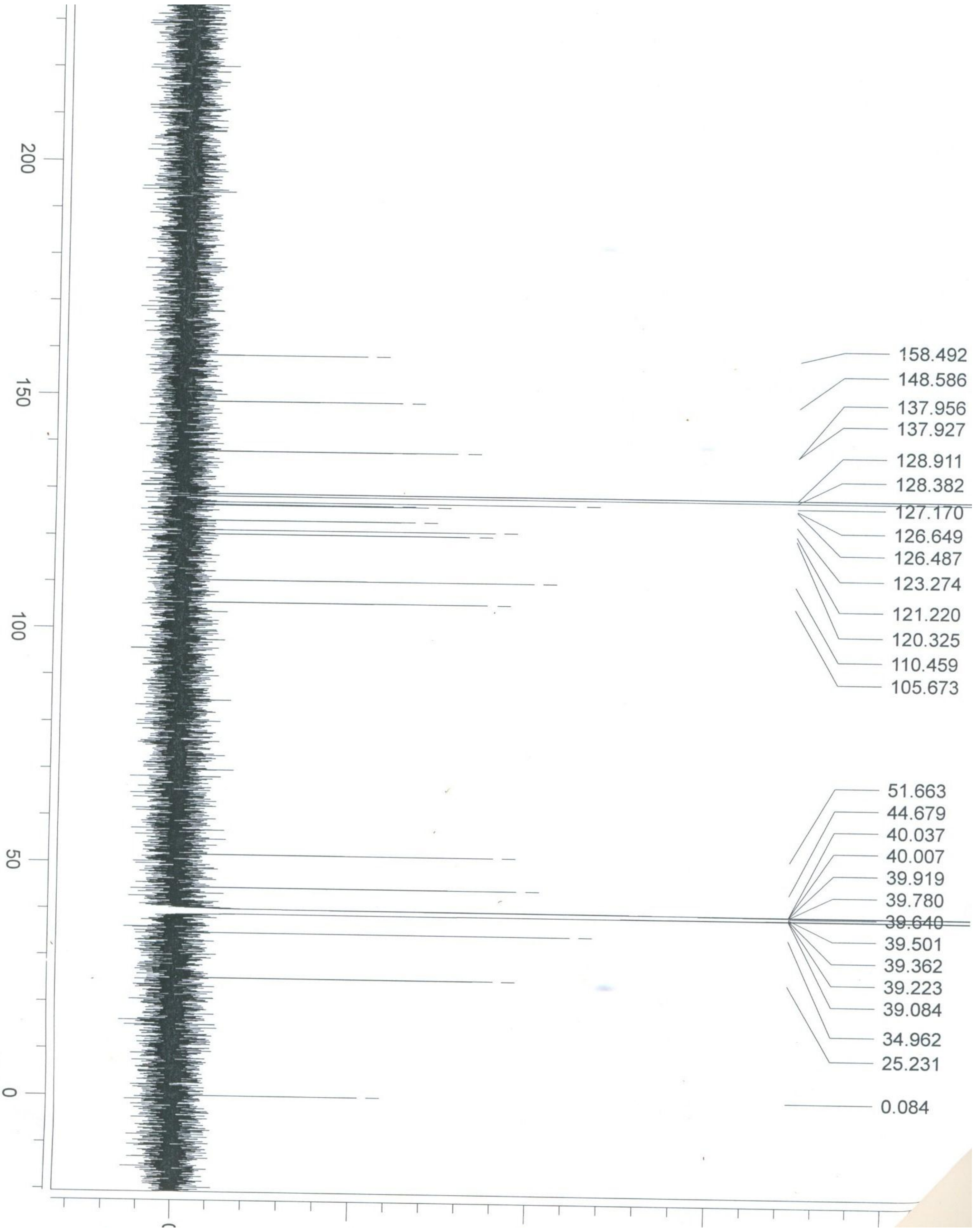

Figure 19. ^{13}C NMR of compound **10**

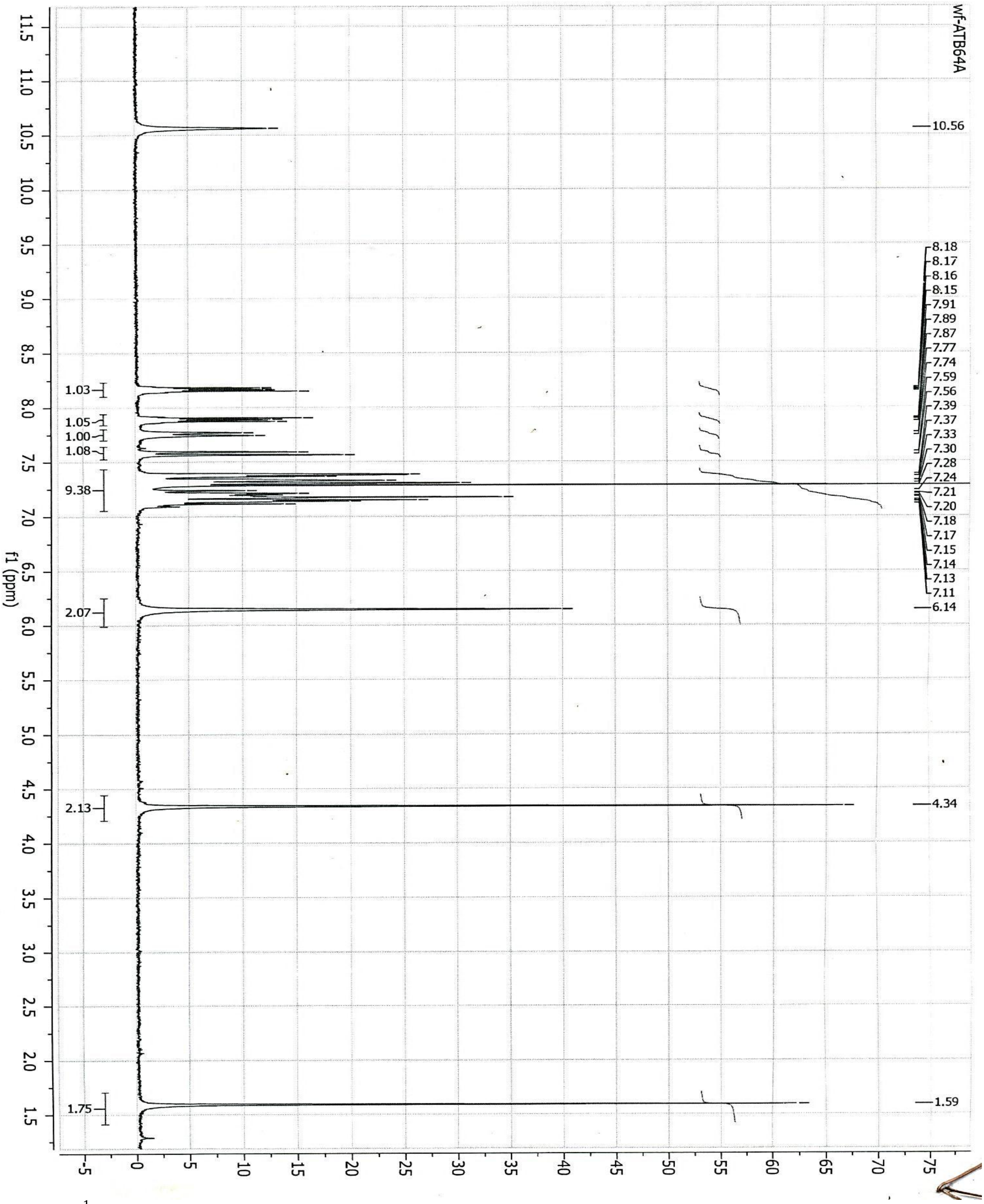

Figure 20. ^{1}H NMR of compound **11**

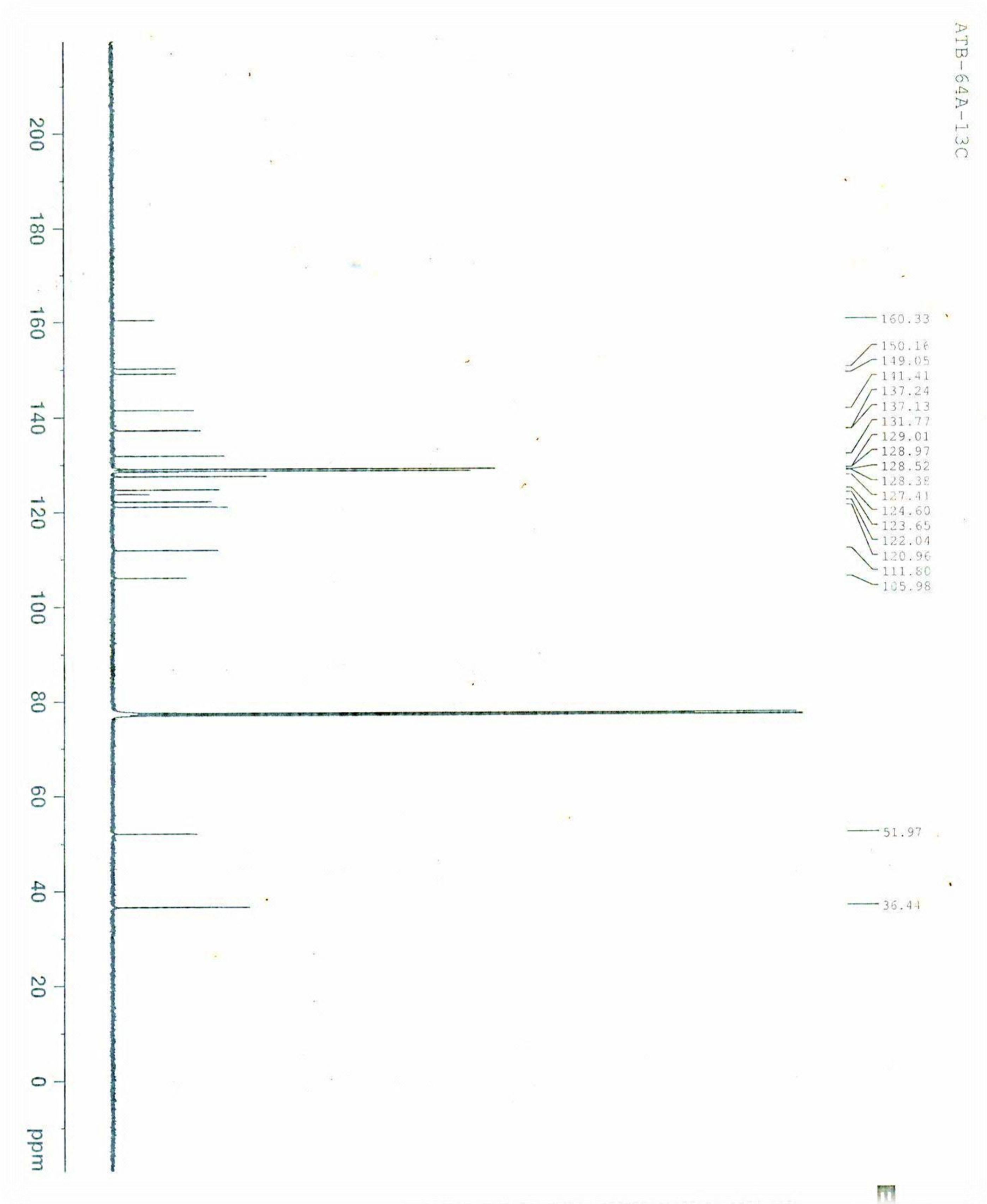

Figure 21. ^{13}C NMR of compound **11**

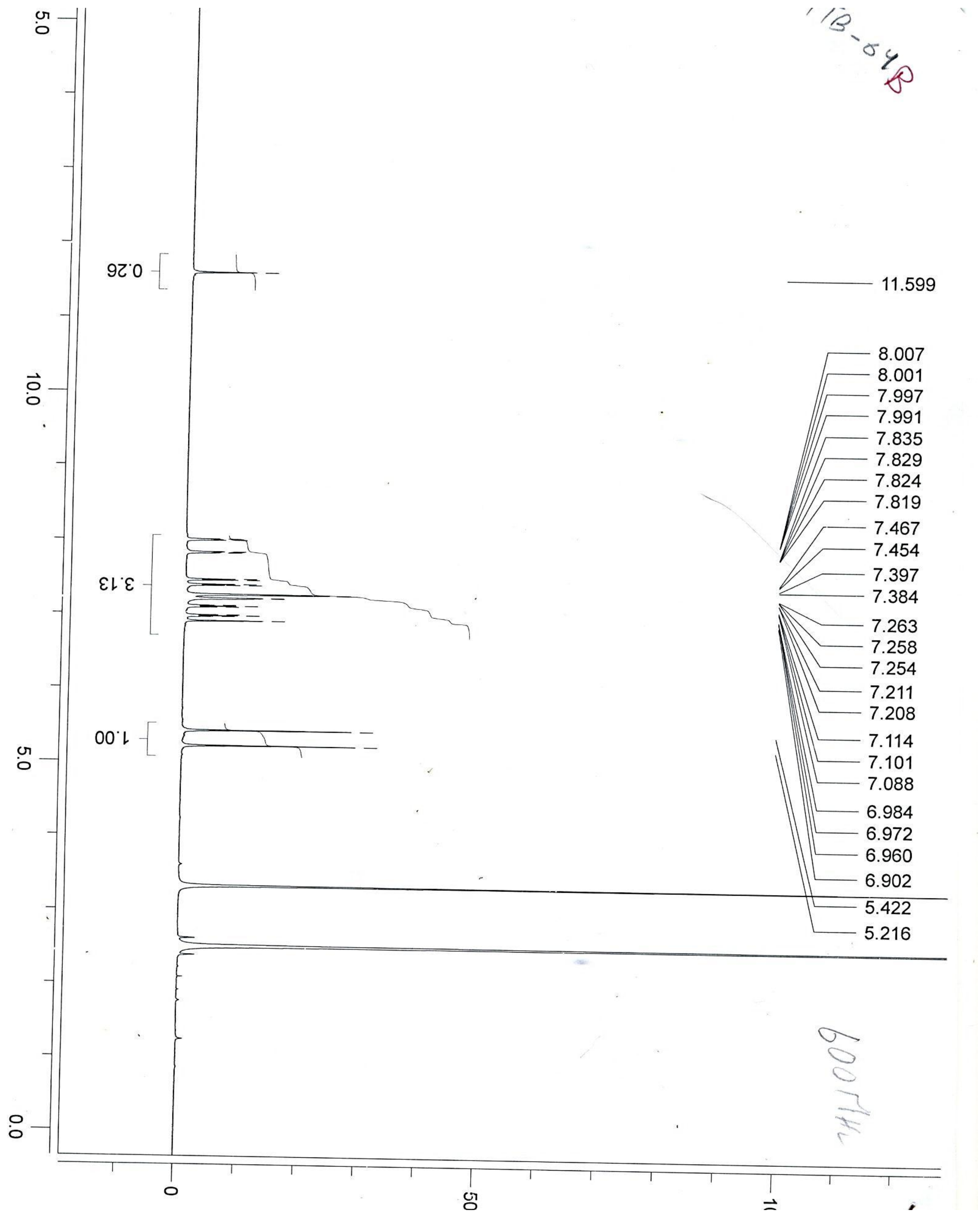

Figure 22. ^{1}H NMR of compound **12**

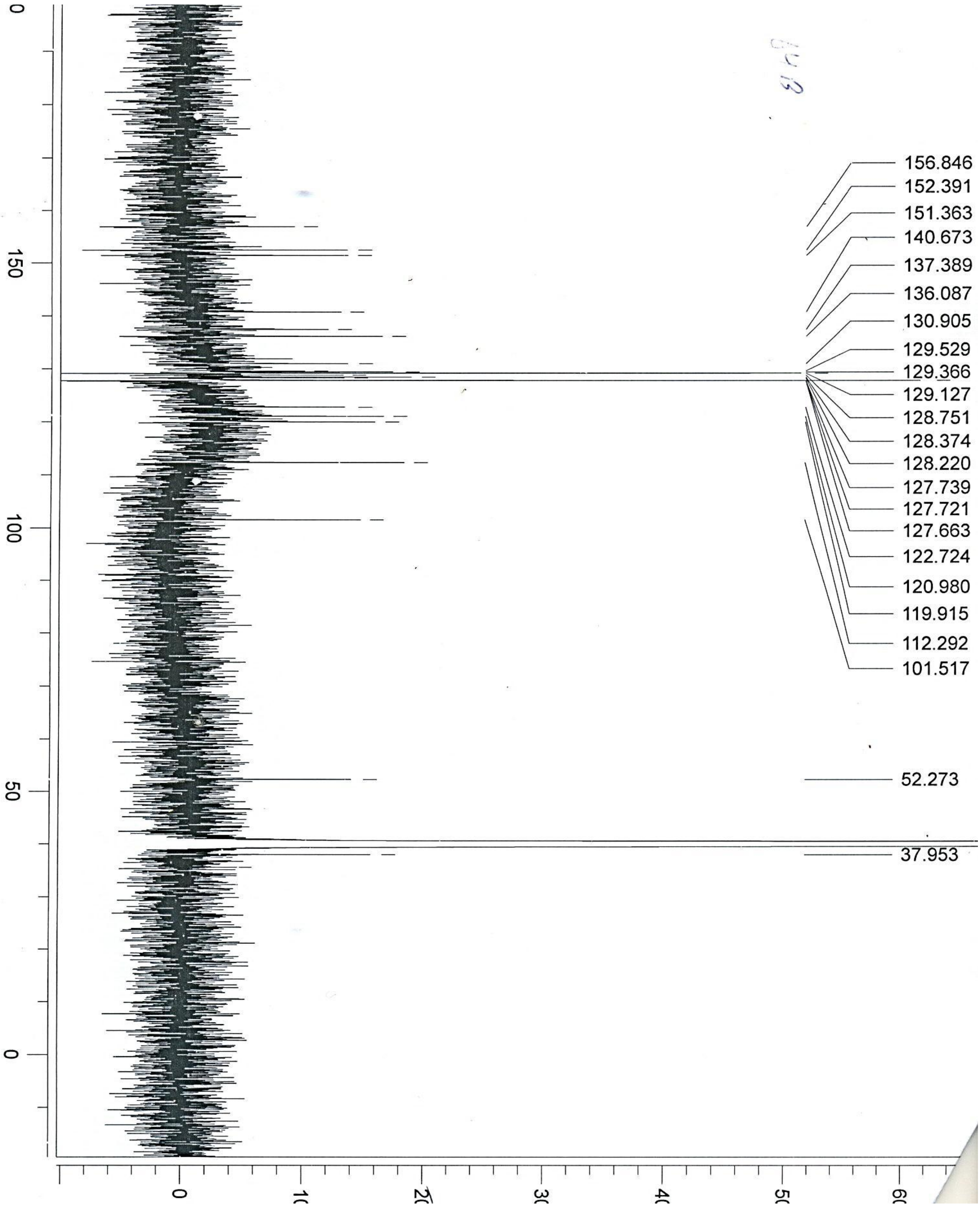

Figure 23. ^{13}C NMR of compound **12**
